# Electronic Effects in a Green Protocol for (Hetero)Aryl-S Coupling

**DOI:** 10.3390/molecules29081714

**Published:** 2024-04-10

**Authors:** Massimo Carraro, Camillo Are, Ugo Azzena, Lidia De Luca, Silvia Gaspa, Giuseppe Satta, Wolfgang Holzer, Vittorio Pace, Luisa Pisano

**Affiliations:** 1 Dipartimento di Scienze Chimiche, Fisiche, Matematiche e Naturali, Università degli Studi di Sassari, Via Vienna 2, 07100 Sassari, Italy; mcarraro@uniss.it (M.C.); ldeluca@uniss.it (L.D.L.); sgaspa@uniss.it (S.G.);; 2 Department of Pharmaceutical Sciences, University of Vienna, Josef-Holaubek-Platz 2, A-1090 Vienna, Austria; wolfgang.holzer@univie.ac.at; 3 Dipartimento di Chimica, Università di Torino, Via Giuria 7, 10125 Torino, Italy; vittorio.pace@unito.it

**Keywords:** Ar-S coupling, CPME, Cu catalysis, electronic effects, green chemistry

## Abstract

Aryl and heteroaryl iodides have been efficiently converted into the corresponding thioacetates in cyclopentyl methyl ether (CPME), a green solvent, under Cu catalysis. The chemoselectivity of the reaction is mainly controlled by electronic factors, enabling the conversion of both electron-rich and electron-deficient substrates into the corresponding thioacetates in good to excellent yields. The products can be easily deprotected to the corresponding thiolates to carry out additional synthetic transformations in situ. Surprisingly, despite CPME’s relatively low dielectric constant, the reaction rate significantly increased when conducted under microwave irradiation conditions. This synthetic methodology exhibits a remarkable tolerance to functional groups, mild reaction conditions, and a wide substrate scope, utilizing a safe and inexpensive CuI pre-catalyst in the green solvent CPME. A non-aqueous workup allowing for the complete recovery of both catalyst and solvent makes this approach an environmentally sustainable protocol for C(sp^2^) sulfur functionalization. Additionally, the reaction shows selective cross-coupling with iodides in competition with chlorides and bromides, allowing its use in multistep syntheses. To demonstrate the potential of this methodology, it was applied to the high-yield synthesis of a photochromic dithienylethene, where a selective synthesis had not been reported before.

## 1. Introduction

**Figure 1:**
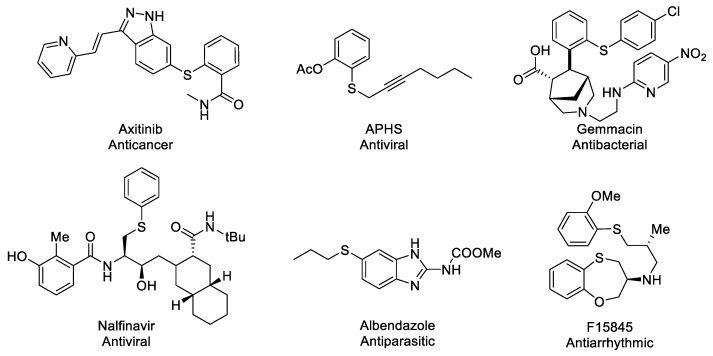
Ar-S-R bioactive molecules.

The C(sp^2^)−SR motif is an important substructure of a variety of biologically active compounds [1,2,3,4,5] (Figure 1). It is also significant in material chemistry, especially in the field of innovative polymeric materials like polyphenylene sulfide (PPS), a special engineering plastic whose applications range from chemical catalysis [6] to optics [7], to nanotechnologies [8], and to the most innovative separation techniques [9]. Moreover, thiols are used in the production of engineering nanomaterials as anchoring groups on noble metals due to the strong affinity of sulfur for these metals surfaces [10,11]. In this context, to prevent the aerobic oxidation of thiols, protected thiols are often used, frequently thioacetates, which are easily cleaved in situ [12].

Accordingly, the construction of Aryl−S bonds is an attractive target in organic chemistry, as it provides useful access to several classes of molecules with a wide range of application fields. Among the numerous methods developed for the introduction of sulfur functions on aromatic and heteroaromatic rings, transition metal-promoted cross-coupling reactions play a central role, with a wide variety of reagents that can be used as sulfur sources [13] and alternative catalytic systems [14]. Pd/, Ni/, and Cu/L complexes are the most employed catalysts [15,16]; however, sustainability concerns have made the use of Cu-based catalysts preferable due to their low costs, low toxicity, the stability of the complexes, and the availability of readily accessible ligands required for the generation of catalytically active systems [17]. In fact, bidentate amines are commonly chosen as ligands for Cu-based catalysts, preferable to the phosphines often used with Pd-based catalysts. Despite the large number of available procedures, synthetic methodologies with optimized green chemistry are extremely rare, and the development of more practical, atomic-economical, and environmentally friendly methods for constructing Ar−S bonds is still a crucial challenge. Potassium thioacetate (AcSK) is a sulfur reagent that offers several advantages. It is classified as non-hazardous, which is beneficial from a safety standpoint. Additionally, AcSK is odorless, stable, and inexpensive, further enhancing its appeal as a sulfur source in chemical reactions. One key advantage of using AcSK is its ability to generate a protected thiol functionality, thus allowing its employment within multistep synthetic projects devoted to the generation of multifunctional structures and requiring further chemoselective transformations [18].

In this regard, in 2013, Peñéñory et al. reported the efficient coupling between aryl iodides and potassium thioacetate, catalyzed by the Cu(I)-1,10-phenanthroline (phen) complex in toluene at 100 °C during 24 h [19]. The mechanism of this reaction has been studied with a multidisciplinary approach, highlighting the preliminary formation of a [Cu(I)/(phen)_2_]^+^ complex which, by reaction with one mole of AcSK, leads to the formation of the catalytically active complex Cu(I)/phen/thioacetate [20]. Finally, an oxidative addition–reductive elimination mechanism via an unstable Cu(III) intermediate was demonstrated. Reactions carried out in other solvents (*N,N*-dimethylformamide, acetonitrile, dimethyl sulfoxide, tetrahydrofuran, and dioxane) led to low or no conversion of the starting material. This confirms the important role played by the solvent in this synthetic transformation, already highlighted from theoretical studies [19,20]. Under similar reaction conditions, the same authors achieved overlapping conversion yields within 2 h by conducting the reactions under microwave irradiation [19]. With the aim of reducing the environmental impact and the cost efficiency of this strategic transformation, we explored the possibility of using CPME as a green solvent and the inexpensive CuI/1,4-diazabicyclo[2.2.2]octane (CuI/DABCO) catalytic system, successfully used in C-S cross-coupling reactions [21]. The closely related and largely employed 2,2′-bipyridine (2,2′-bipy) was also tested as a cheap Cu(I) ligand. Actually, CPME is industrially produced from non-renewable sources with a 100% atom-economical and high-yield process by Zeon Corporation [22]. Its chemical–physical properties, such as a wide liquidity range, hydrophobicity, low heat of vaporization, chemical stability, and resistance to peroxide formation, make it a safer and low-environmental-impact solvent [23,24]. One key advantage of CPME is its low energy consumption during the recovery phase. Furthermore, CPME is not mutagenic or genotoxic and shows low acute and subchronic toxicity [25]. Its use has increased in both academia and industry in recent years; this could make it economically competitive, which is one of the few factors negatively affecting its sustainability [24]. Among other applications, CPME has been extensively employed in transition metal-catalyzed cross-coupling reactions [24], although, to the best of our knowledge, it has never been employed under Cu/L-catalysis.

This work based on Cu-catalyzed C(sp^2^)-S cross-coupling reactions is focused on improvements in cost efficiency and environmental impact and exploring their versatility in multistep synthesis.

## 2. Results and Discussion

We began our investigations on the C(sp^2^)-S coupling of (hetero)aryl halides with AcSK in CPME by employing phenyl iodide and phenyl bromide as substrates and CuI as a pre-catalyst along with 1,10-phenantroline (phen), 2,2′-bipy, or DABCO as nitrogen bidentate ligands. In the first reaction, iodobenzene **1** reacted, under argon atmosphere, with 1.5 eq. of AcSK in the presence of 0.1 eq. of the [Cu(I)/(phen)_2_]^+^ complex in CPME for 24 h at 100 °C to afford the corresponding thioacetate derivative **4** in an almost quantitative yield (Table 1, entry 1). Lower yields were achieved by either reducing the catalyst loading (Table 1, entry 2) or reducing the reaction time (Table 1, entry 3). Under the same conditions, no reaction was observed with bromobenzene **2** (Table 1, entry 4), revealing the complete selectivity of the reaction for aryl iodides in accordance with observations from other groups [19,26]; this finding agrees with oxidative addition as a rate-determining step, since C−Br bond breaking involves higher activation energy.

The reaction carried out using bromobenzene did not lead to the formation of the desired product even with an extended reaction time of 48 h, under reflux conditions, or when the quantity of catalyst and ligand was doubled. Furthermore, when CPME was substituted with more polar solvents like dimethylformamide or acetonitrile, bromobenzene did not undergo the desired reaction.

Despite a narrower substrate scope, selectivity for aryl iodides can be advantageous for further synthetic transformations [27].

To improve the cost efficiency of the reaction, *N,N*-bidentate ligands DABCO and 2,2′-bipy were used, but both led to low yields with phenyl iodide **1** (Table 1, entries 6 and 8) and no reaction with the Br-derivative **2** (Table 1, entry 7 and 9). To assess the versatility of the reaction toward polyfunctional molecules, we performed the reaction on the diiodo-derivative **3**, obtaining the double functionalization in the presence of doubled amounts of reagents (Table 1, entry 5) in excellent yield. Lower yields were observed if the reactions were carried out in air atmosphere or reducing temperature.

The efficiency of the reaction was therefore evaluated by extending the procedure to different (hetero)aryl iodides (Table 2), by using the [Cu(I)/(phen)_2_]^+^ complex as a catalyst. Based on the previously described mechanism, we explored the effect of the substrates’ electronic properties on the reactivity. As already reported, Cu-catalyzed cross-coupling reactions between aryl iodides and potassium thioacetate are seriously affected by the solvent employed. In detail, the reaction which successfully runs in toluene seems to be negatively affected by the use of polar solvents, both in terms of reactivity and chemoselectivity [19]. To address this issue, an investigation was conducted to assess the outcome of these reactions using CPME, which was still characterized by low polarity but was slightly higher compared to toluene. Additionally, the investigation explored the use of conventional heating or possibly microwave irradiation (reported in the row below in Table 2) as alternative reaction conditions. These different heating methods can further affect the reaction kinetics and potentially enhance the efficiency of Cu-catalyzed cross-coupling reactions.

The data in Table 2 show that using CPME as a solvent, despite its higher polarity than toluene, preserves the reactivity and chemoselectivity of the reaction [19]. Like iodobenzene **1a**, all the electron-neutral derivatives led exclusively to the formation of the corresponding thioacetyl derivatives. The 4-thioacetyl-derivative of 1,1′-biphenyl (**4b**, Table 2, entry 2) was obtained in an almost quantitative yield, while lower yields were obtained with the sterically hindered derivatives **1c** (64%) and **1d** (40%) (Table 2, entries 3 and 4). On the contrary, 4-chloroiodobenzene **1e**, besides disclosing complete chemoselectivity for iodide over chloride, provided the expected product with almost quantitative conversion (Table 2, entry 5). Even in the absence of steric effects, the electron-rich substrate **1f** did not react quantitatively but led to the exclusive formation of the desired product (Table 2, entry 6). The most electron-poor substrates **1g**–**1k** showed greater reactivity but lower chemoselectivity compared to the electron-rich ones; indeed, in addition to the desired products **4g**–**4k**, the formation of small amounts of the corresponding diaryl sulfides **6g**–**6k** was also obtained (Table 2, entries 7–11). Noteworthily, the chemoselectivity appeared to be correlated with the electron density of the aromatic ring. Indeed, substrates bearing more electron-withdrawing substituents led to the formation of a greater amount of the corresponding by-product **6**, while substrates with moderate electron-withdrawing substituents, such as **1e** and **1l**, exhibited high selectivity and led exclusively to the formation of the desired products (Table 2, entries 5 and 12). Finally, the reactivity of some heteroaromatic iodides was also investigated (Table 2, entries 13–15). 2-Iodothiophene **1m** and the corresponding 3-substituted isomer **1n** reacted chemoselectively, leading to the corresponding thioacetyl derivative **4m** and **4n** with good yields (Table 2, entries 13 and 14). These results are consistent with the electron-rich nature of the thiophenic ring and show a very small difference in the electron density of positions 2 and 3 of the heteroaromatic ring.

The unexpected result observed in the case of 2-iodopyridine **1o** (Table 2, entry 15) deserves a separate comment. The pyridinic ring is an electron-deficient heteroaromatic, and, based on the previous results, one might have expected high conversion and low chemoselectivity. However, as previously described in the literature [28], in the Cu(I)-catalyzed coupling of aryl halides with sulfur-based nucleophiles, pyridinic derivatives can compete with 1,10-phen as ligands. This competition between ligands can affect the outcome of the reaction, influencing factors such as reaction rate, selectivity, and the stability of the formed complexes.

Under microwave irradiation, an important kinetic effect was observed in CPME (Table 2, entries 1, 6, 8–10), regardless of the electronic properties of the substrates, comparable to what has been previously reported for reactions carried out in toluene [19]. However, this did not significantly affect the conversion or selectivity of the reactions.

**Scheme 1:**
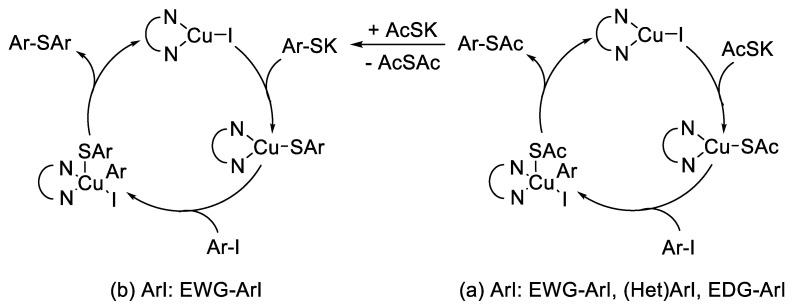
Plausible reaction mechanism for the Cu^I^−phen-catalyzed Ar−SAc coupling (**a**); plausible reaction mechanism for the Cu^I^−phen-catalyzed Ar−S−Ar coupling (**b**).

Taken together, these findings support the previously proposed mechanism where the rate-determining step, an oxidative addition to give a Cu(III) intermediate, can be promoted by electron-poor (hetero)aryl halides [20]. Furthermore, electron-deficient substrates, besides showing greater reactivity, lead to the formation of the corresponding diaryl sulfides as by-products of the reactions (Table 2, entries 7–11 and 13). The competitive Cu-catalyzed cross-coupling reaction between aryl iodides **1** and aryl thiolates already described in the literature [26,29,30,31] probably takes place as the concentration of the aryl thiolate increases in the reaction medium, which occurs selectively with the aryl thioacetates, whose aryl thiolate is stabilized by electronic effects. Probably, as already observed for the ammonolysis of aryl thioacetates [32], the electron-poor aryl thioesters undergo faster deacetylation at the sulfur atom, thus generating the aryl thiolates, being able to compete with the AcS^−^ anion in the catalytic cycle [33]. The overall reactivity observed can be explained by proposing that, for electron-rich or electron-neutral compounds, the previously described catalytic cycle operates [20], whereas for electron-poor substrates, there is competition with a secondary catalytic cycle. This secondary cycle involves the formation of the Cu(I)−phen−SAr complex, which undergoes the oxidative addition of the substrate and the reductive elimination of the symmetrical diaryl sulfide, as reported in Scheme 1. Unfortunately, this side reaction could be promoted by the excess of AcSK necessary for the successful outcome of the reaction within a reasonable time.

**Scheme 2:**
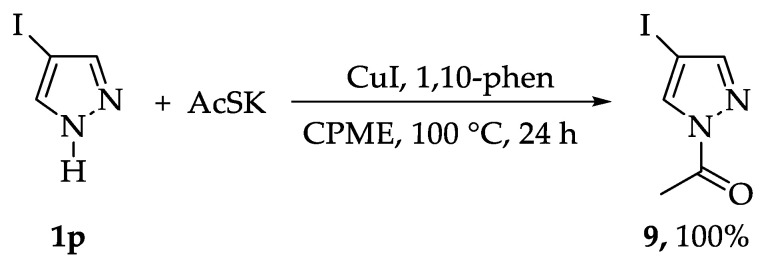
Acyl transfer on the heteroaryl iodides **1p**.

In addition to those reported in Table 2, a further substrate was taken into consideration, 4-iodopyrazole **1p**. Under our reaction conditions, this substrate underwent a transformation which involved not coupling with the heteroaryl iodide function, but rather the nucleophilic nitrogen atom, as reported in Scheme 2. Probably, the nitrogen nucleophilicity promotes acyl transfer from the catalytically active complex Cu(I)/phen/thioacetate and inhibits the cross-coupling reaction on the heteroaryl iodide.

Even if the reaction is performed in the presence of an excess of AcSK (three equivalents), the selective formation of the *N*-acylation product was observed. However, in this case, the reaction proceeded with a lower conversion yield, leading to a mixture of the *N*-acylated derivative, heteroaryl iodide, and free 1,10-phenanthroline.

To improve the sustainability of the process, we also optimized the workup procedure. Indeed, after the reaction, products and possibly the substrates or diaryl sulphides (depending on the reactivity and chemoselectivity) were dissolved in CPME, while the Cu−phen catalytic complex and KI were insoluble. Therefore, the simple filtration of the reaction mixture allowed us to isolate the products which, if necessary, could be further purified by flash chromatography. Thus, the recovery of the reaction solvent, the only one used in the procedure, became almost quantitative. A further step toward the sustainability of the process includes the recovery of the catalytic system, which is easily recoverable and recyclable, as already reported [34]. In our experimental conditions, the catalytic complex was isolated through straightforward filtration, dried, and reused without additional processing. Despite containing the by-product KI, the resulting complex was reused three times without the significant loss of catalytic activity. However, it should be noted that normally, the synthesis of thioacetates is functional to the generation of precursors of molecules bearing the arylthiolic group, which can be released in situ during the final transformation [35]. This last step can be carried out under extremely mild basic conditions [36] and leads to the formation of the corresponding aryl thiolates, which can be trapped in situ with electrophilic reagents [19].

**Scheme 3:**
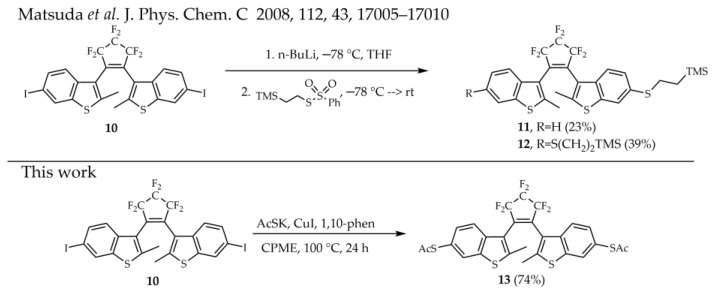
Synthesis of the protected photochromic dithienylethene **13** [37].

The methodology has been successfully applied to the synthesis of the acetyl-protected 3,3′-(perfluorocyclopent-1-ene-1,2-diyl)bis(2-methylbenzo[b]thiophene-6-thiol) **13**, a photochromic thienyl-type diarylethene with interesting applications in molecular electronics [37,38]. Despite its photochemical properties, the selective synthesis of this molecule has never been reported. Indeed, in 2008, Irie and colleagues, studying photoswitching behavior on the conductance of diarylethene units linked to gold [37] and silver [38] nanoparticles, synthetized the trimethylsilylethyl-protected photochromic dithienylethene **11**, starting from the diiododerivative **10** [39] with low selectivity and under harsh conditions (Scheme 3). With our methodology, we performed Ar−S coupling starting from the same substrate, obtaining the acetyl-protected derivative **13** with high yield and selectivity under mild reaction conditions (Scheme 3).

## 3. Materials and Methods

### 3.1. Materials

All the reactions were carried out under the inert atmosphere of argon. CPME was distilled over Na/benzophenone under argon. Chemicals were obtained from commercial suppliers and used without further purification. Column chromatography was performed on silica gel (pore size 60 Å, 32–63 nm particle size), and reactions were monitored by thin-layer chromatography (TLC) analysis using Merck (Macherey-Nagel, Dueren, Germany) Kieselgel 60 F254 plates, and visualization was achieved under UV light at 254 nm. Solutions were evaporated under reduced pressure with a rotary evaporator.

The ^1^H NMR and ^13^C NMR spectra (500 MHz and 125 MHz, respectively) were recorded on a Bruker Avance 500 Ultrashield spectrometer (Bruker, Ettlingen, Germany) at 297 K, using CDCl_3_ signals as an internal standard. Chemical shifts are reported in parts per million (ppm, δ), calibrated using residual non-deuterated solvent as an internal reference (CHCl_3_ at δ 7.26 ppm (^1^HNMR) and at δ 77.16 ppm (^13^CNMR)). The peak patterns are indicated as follows: s, singlet; d, doublet; t, triplet; m, multiplet; q, quartet; dd, doublet of doublets; br, broad. The coupling constants, J, are reported in Hertz (Hz). In nearly all cases, the full and unambiguous assignment of all resonances was performed by the combined application of standard NMR techniques, such as ^1^HNMR, ^13^CNMR, ^13^C-APT, HSQC, and ^19^FNMR experiments. Mass spectra were obtained on a Bruker maXis 4G instrument (ESI-TOF, HRMS) (Bruker, Bremen, Germany). The microwave reactions were carried out by using an MC8S-3 microwave instrument.

### 3.2. General Procedure for the Synthesis of the Substrates ***4a***–***4o***, ***5***, and ***9***

The reactions were carried out in a 25 mL two-necked flask, equipped with a magnetic stirrer, a condenser, and an argon inlet.

To a stirring solution of ArI (0.5 mmol; 1 eq) in CPME (2 mL) the following were added in the following order: CuI (0.01 g; 0.05 mmol, 0.1 eq), ligand (0.1 mmol, 0.2 eq, 1,10-phenanthroline, 0.02 g; DABCO, 0.012 g; or 2,2′-bipyridine, 0.016 g) and potassium thioacetate (0.09 g, 0.75 mmol, 1.5 eq). The reaction mixture was warmed to 100 °C and stirred at this temperature for 24 h under argon before being cooled to room temperature. The catalytic complex was separated by filtration, dried under reduced pressure, and reused without further elaboration. The solvent was evaporated under reduced pressure from the filtered CPME solution containing the reaction mixture and recycled. If necessary, the chromatographic purification of the product was performed.

### 3.3. General Procedure for the Synthesis of Substrates ***4a***, ***4f***, and ***4h***–***4j*** under Microwave Irradiation

The same reactions were carried out in a 10 mL microwave tube under argon atmosphere and irradiated (starting output power: 300 W) whilst stirring at 100 °C for 2 h (1 cycle).

### 3.4. Synthesis of 3,3′-(Perfluorocyclopent-1-ene-1,2-diyl)bis(2-methyl-6-(acetylthio)-benzo[b]thiophene) ***13***

A 100 mL two-necked Schlenk tube, equipped with an argon gas inlet, a condenser, and a magnetic stirrer, was dried under vacuum, filled with argon, and then charged with dried cyclopentyl methyl ether (CPME) (20 mL). 1,2-Bis(6-iodo-2-methyl-1-benzothiophen-3-yl)hexafluorocyclopentene **10** (1.08 g, 1.45 mmol), CuI (10 mol%), 1,10-phenanthroline (20 mol%), and finally, potassium thioacetate (2.17 mmol, 1.5 eq.) were added under argon and stirred at 100 °C for 18 h, monitored by TLC (cyclohexane/AcOEt = 9:1, Rf = 0.153). The reaction mixture was cooled to room temperature and quenched with water (20 mL). The organic layer was separated and the aqueous layer was extracted with CPME (3 × 20 mL). The combined organic extracts were dried over CaCl_2_, and the crude reaction mixture was purified by silica gel column chromatography (gradient 100% cyclohexane–cyclohexane/AcOEt = 8.5:1.5), giving 0.68 g of 3,3′-(perfluorocyclopent-1-ene-1,2-diyl)bis(2-methyl-6-(acetylthio)benzo[b]thiophene) **13** (1.10 mmol, yield 76%) as a mixture of conformational isomers (parallel/antiparallel = 35:65).

## 4. Conclusions

An eco-friendly, efficient methodology was developed for C(sp^2^)−S coupling in CPME under conventional heating or microwave irradiation, promoted by the in situ generated [Cu/(1,10-phen)_2_] as a cheap and easily recyclable catalyst. Under our reaction conditions, complete selectivity was observed for Ar−I with respect to Ar−Cl and Ar−Br, thus allowing further synthetic transformations. According to the proposed mechanism, both steric and electronic effects dominated the reaction. Excellent reactivity and selectivity were exclusively observed with electron-neutral substrates, while electron-poor substrates, though highly reactive, showed poor chemoselectivity. On the other hand, electron-rich substrates, while less reactive, produced the desired products with complete selectivity. The overall reactivity is explained by a secondary catalytic cycle that involves the formation of the Cu(I)−phen−SAr complex, specifically active with electron-poor substrates. Remarkably, this rationalization allows for the prediction of the reaction outcomes beforehand. The tolerance to functional groups is limited by the presence of strongly nucleophilic functional groups in the substrates, which can promote an acyl transfer process, compromising the cross-coupling reaction. To enhance the sustainability of the synthetic methodology, a non-aqueous workup was developed, enabling the recycling of the catalytic complex and quantitative recovery of the solvent.

Lastly, the efficiency and versatility toward polyfunctional molecules were demonstrated by the selective and high-yielding synthesis of 3,3′-(perfluorocyclopent-1-ene-1,2-diyl)bis(2-methylbenzo[b]thiophene-6-thiol) acyl-protected 13, a photochromic thienyl-type diarylethene with interesting applications in molecular electronics.

## Figures and Tables

**Table 1:** The screening of ligands and substrates for the Cu/L-catalyzed coupling of AcSK with haloaryls in CPME ^1^.

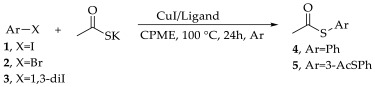				
Entry ^1^	Ar-X	Ligand	Product	Yield (%) ^2^
1	Ph-I (**1**)	1,10-phen	**4**	98
2	Ph-I (**1**)	1,10-phen	**4**	30 ^3^
3	Ph-I (**1**)	1,10-phen	**4**	56 ^4^
4	Ph-Br (**2**)	1,10-phen	-	-
5	*m*-I_2_C_6_H_4_ (**3**)	1,10-phen	**5**	87 ^5^
6	Ph-I (**1**)	DABCO	**4**	15
7	Ph-Br (**2**)	DABCO	-	-
8	Ph-I (**1**)	2,2′-Bipy	**4**	10
9	Ph-Br (**2**)	2,2′-Bipy	-	-

^1^ Reaction conditions: Ar−X (2 mmol), potassium thioacetate (3 mmol), CuI (0.2 mmol), ligand (0.4 mmol), CPME (8 mL) 100 °C, 24 h under argon. ^2^ Isolated yields. ^3^ Reaction performed with 0.05 eq of CuI and 0.1 eq of ligand. ^4^ Reaction performed for 12 h. ^5^ Reaction performed with double amounts of reagents; 10% of 3-iodophenyl thioacetate was also obtained.

**Table 2:** Electronic effects in C_(Het)Ar_-S cross-coupling reactions catalyzed by Cu(I)/(1,10-phen)_2_ complex.

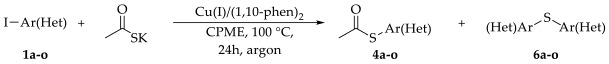				
Entry ^1^	Ph-X(1)	Conversion	Product	Entry ^1^
		(%) ^2^	4(%) ^2^	6(%) ^2^
1	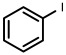 **1a**	100	100 (98)	-
		100 ^3^	100 ^3^	-
2	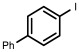 **1b**	94	100 (90)	-
3	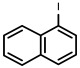 **1c**	74	100 (71)	-
4	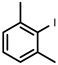 **1d**	40	100 (36)	-
5	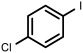 **1e**	97	100 (94)	-
6	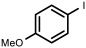 **1f**	90	100 (87)	-
		88 ^3^	100 ^3^	-
7	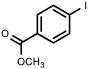 **1g**	100	93	7
8	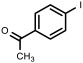 **1h**	100	92 (87)	8
		100 ^3^	79 ^3^	21 ^3^
9	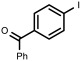 **1i**	100	90 (87)	10
		100 ^3^	85 ^3^	15 ^3^
10	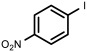 **1j**	100	73	27
		100 ^3^	70 ^3^	30^3^
11	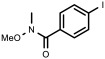 **1k**	100	81 (76)	19
12	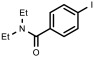 **1l**	66	100 (62)	-
13	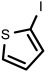 **1m**	88	100 (88)	-
14	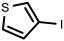 **1n**	80	100 (72)	-
15	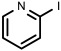 **1o**	60	100 (57)	-

^1^ Reaction conditions: CuI (0.1eq, 0.05 mmol); 1,10-phen (0.2eq, 0.1 mmol), AcSK (0.75 mmol), ArI (0.5 mmol); CPME (2 mL), 100 °C, 24 h, under argon. ^2^ Conversion yields and products ratio determined by ^1^H-NMR analysis of reaction mixtures; isolated yields are reported in brackets. ^3^ Reaction performed under microwave irradiation; reaction conditions: 1 cycle of 2 h, 100 °C, 300 W.

## Data Availability

The data presented in this study are available in the Supplementary Materials.

## Supplementary Materials

The following supporting information can be downloaded at https://www.mdpi.com/article/10.3390/molecules29081714/s1, General information; General procedure for the synthesis of the substrates **4a**–**4o**, **5** and **9**; General procedure for the synthesis of the substrates **4a**, **4f**, and **4h**–**4j** under microwave irradiation; Characterization data: Product **4a**–**4o**, **5**, and **9**; Synthesis of 1,2-Bis(6-iodo-2-methyl-1-benzothiophen-3-yl)hexafluorocyclopentene **10**; Characterization data: Product **10**; Synthesis of 3,3′-(perfluorocyclopent-1-ene-1,2-diyl)bis(2-methyl-6-(acetylthio)benzo[b]thiophene) **13**; Characterization data: Product **13**. Refs. [40,41,42,43,44] have been cited in Supplementary Materials.
