# Supplementary material for: Electronic Effects in a Green Protocol for (Hetero)Aryl-S Coupling
Source: Molecules. 2024 Apr 10;29(8):1714. doi: 10.3390/molecules29081714 (PMC11051792; doi:10.3390/molecules29081714)
Supplement: Supplementary file 1 [file molecules-29-01714-s001.zip › molecules-2914641-supplementary.pdf]

Supporting Information

Electronic Effects in a Green Protocol for (Hetero)Aryl-S Coupling

Massimo Carraro,[1] Camillo Are,[1] Ugo Azzena,[1] Lidia De Luca,[1] Silvia Gaspa,[1] Giuseppe Satta,[1] Wolfgang Holzer,[2] Vittorio Pace,[3] and Luisa Pisano[1]

[1] Dipartimento di Scienze Chimiche, Fisiche, Matematiche e Naturali, Università di Sassari, via Vienna 2, 07100 Sassari, Italy
[2] Department of Pharmaceutical Sciences, University of Vienna, Josef-Holaubek-Platz 2, A-1090 Vienna, Austria
[3] Dipartimento di Chimica, Università di Torino, Via Giuria 7, I-10125 Torino, Italy
* Correspondence: luisa@uniss.it

Table of contents

Instrumentation and General Analytical Methods	S2
General procedure for the synthesis of the substrates **4a-4o, 5** and **9**	S2
General procedure for the synthesis of the substrates **4a**, **4f**, **4h-4j** under microwave irradiation	S2
Characterization Data: Product **4a-4o**, **5** and **9**	S3-S19
Synthesis of 1,2-Bis(6-iodo-2-methyl-1-benzothiophen-3-yl)hexafluorocyclopentene (**10**)	S20
Characterization Data: Product **10**	S20-S22
Synthesis of 3,3'-(perfluorocyclopent-1-ene-1,2-diyl)bis(2-methyl-6-(acetylthio)benzo[b]thiophene) (**13**)	S22
Characterization Data: Product **13**	S23-S25

Instrumentation and General Analytical Methods

^{1}H NMR and ^{13}C NMR spectra were recorded on a Bruker Avance 500 Ultrashield spectrometer (500 MHz and 125 MHz, respectively) at 297 K, using $CDCl_3$ signals as an internal standard. Chemical shifts are reported in parts per million (ppm, δ) calibrated using residual non deuterated solvent as internal reference ($CHCl_3$ at δ 7.26 ppm(^{1}HNMR) andδ 77.16 ppm(^{13}C NMR)). The peak patterns are indicated as follows: s, singlet; d, doublet; t, triplet; m, multiplet; q, quartet; dd, doublet of doublets; br, broad. The coupling constants J, are reported in Hertz (Hz). In nearly all cases, full and unambiguous assignment of all resonances was performed by combined application of standard NMR techniques, such as ^{1}HNMR, ^{13}CNMR, ^{13}C-APT, HSQC and ^{19}FNMR experiments. Mass spectra were obtained on a Bruker maXis 4G instrument (ESI-TOF, HRMS). The microwave reactions were carried out by using MC8S-3 microwave instrument. All the reactions were carried out under inert atmosphere of argon. CPME was distilled over Na/benzophenone under argon. Chemicals were purchased from Sigma-Aldrich, Acros, Alfa Aesar and TCI Europe. Column chromatography was performed on silica gel (pore size 60 Å, 32- 63 nm particle size) and reactions were monitored by thin-layer chromatography (TLC) analysis using Merck (Macherey-Nagel) Kieselgel 60 F254 plates and visualized with UV light at 254 nm. Solutions were evaporated under reduced pressure with a rotary evaporator.

General procedure for the synthesis of the substrates 4a-4o, 5 and 9.

The reactions were carried out in a 25 mL two necked flask, equipped with a magnetic stirrer, a condenser and an argon inlet.

To a stirring solution of ArI (0.5 mmol; 1eq) in CPME (2mL) were added in the following order: CuI (0.01 g; 0.05 mmol; 0.1 eq), ligand (0.1 mmol; 0.2 eq)* and potassium thioacetate (0.09g; 0.75 mmol; 1.5 eq). The reaction mixture was warmed to 100 °C and stirred at this temperature for 24 h under argon before being cooled to room temperature. The catalytic complex was separated by filtration, dried under reduced pressure, and reused. The solvent was evaporated under reduced pressure from the filtered CPME solution containing the reaction product and recycled. If necessary, chromatographic purification was performed.

Apart for **4a** and **9**, all the products were purified by column chromatography on silica gel.

*1,10-phenanthroline, 0.02 g; DABCO, 0.012 g; or 2,2'-bipyridine, 0.016 g.

General procedure for the synthesis of the substrates 4a, 4f, 4h-4j under microwave irradiation

The same reactions were carried out in a 10-mL microwave tube under argon atmosphere, and irradiated (starting output power: 300 W) whilst stirring at 100 °C for 2h (1cycle).

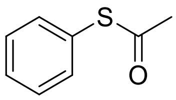

***S*-phenyl ethanethioate (4a).** Brown oil; ^{1}H NMR (500 MHz, $CDCl_3$) δ (ppm) 7.42 (s, 5H, aromatic) 2.45 (s, 3H, CH_3); ^{13}C NMR (125 MHz, $CDCl_3$) δ (ppm) 194.09 (COS), 134.44 (Ph C-2,6), 129.43 (Ph C-4) 129.19 (Ph C-3,5), 127.87 (Ph C-1), 30.19 (CH_3); HRMS (ESI) *m/z*: calc. for C_8H_8NaOS: 175.0195 $[M+Na]^+$, found: 175.0188; R_f = 0.41 (*n*-hexane/EtOAc = 9:1); isolated yield= 98%.

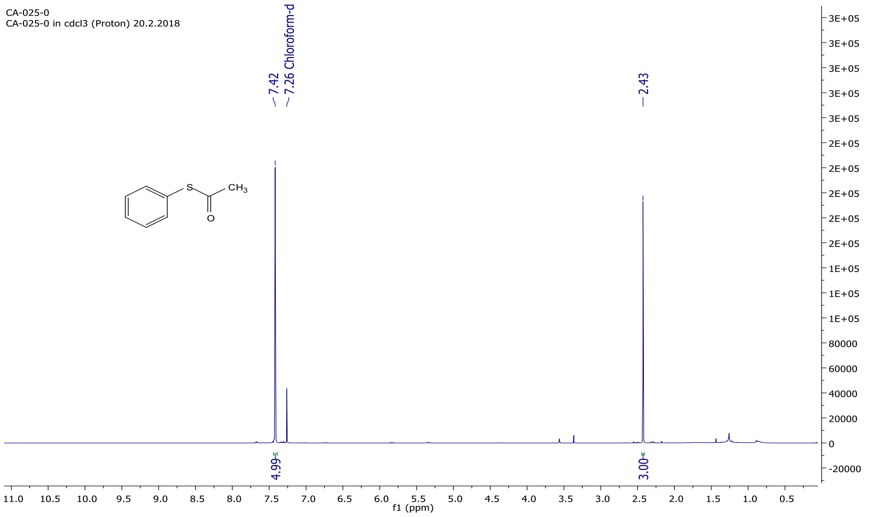

Figure S1 – ^{1}H NMR spectrum of **4a**

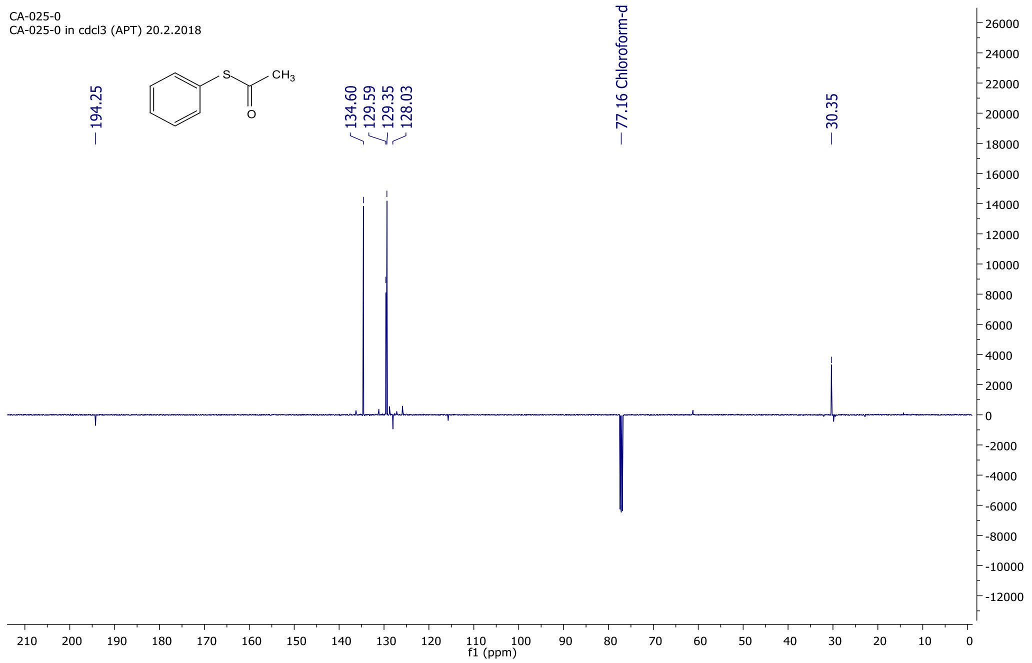

Figure S2 – ^{13}C NMR spectrum of **4a**

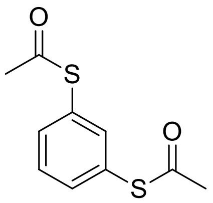

***S,S'*-1,3-phenylene diethanethioate (5).** Red oil; ^{1}H NMR (500 MHz, $CDCl_3$) δ (ppm) 7,48 (m, 1H, Ph H-2), 7.46 (m, 1H, Ph H-5), 7.45 (m, 2H, Ph H-4,6), 2.43 (s, 3H, CH_3); ^{13}C NMR (125 MHz, $CDCl_3$) δ (ppm) 193.32 (COS), 139.79 (Ph C-2), 135.31 (Ph C-4,6), 129.78 (Ph C-5), 129,04 (Ph C-1,3), 29.49 (CH_3); HRMS (ESI) *m/z*: calc. for $C_{10}H_{10}NaO_2S_2$: 249.0019 $[M+Na]^+$, found: 249.0014; R_f = 0.29 (*n*-hexane/EtOAc = 9:1); isolated yield: 87%

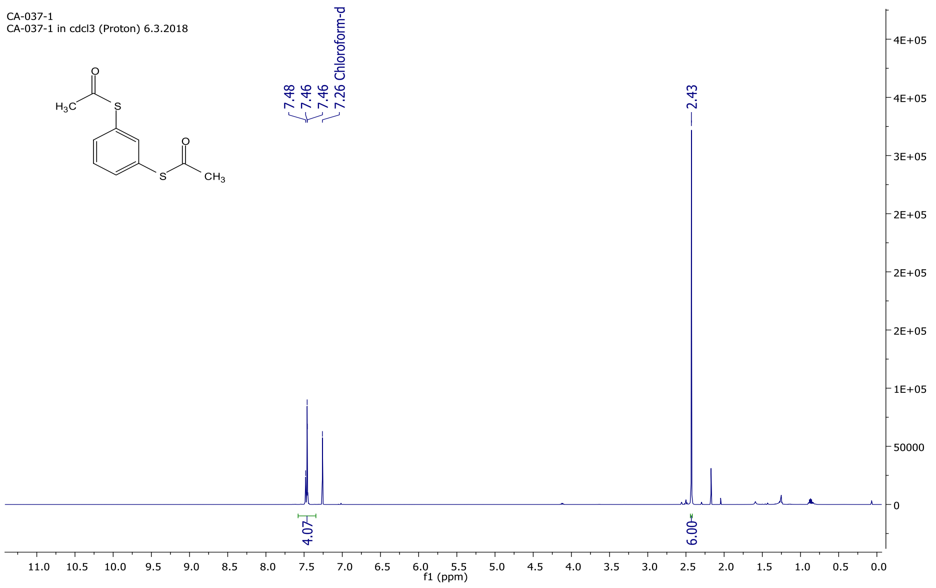

Figure S3 – ^{1}H NMR spectrum of **5**

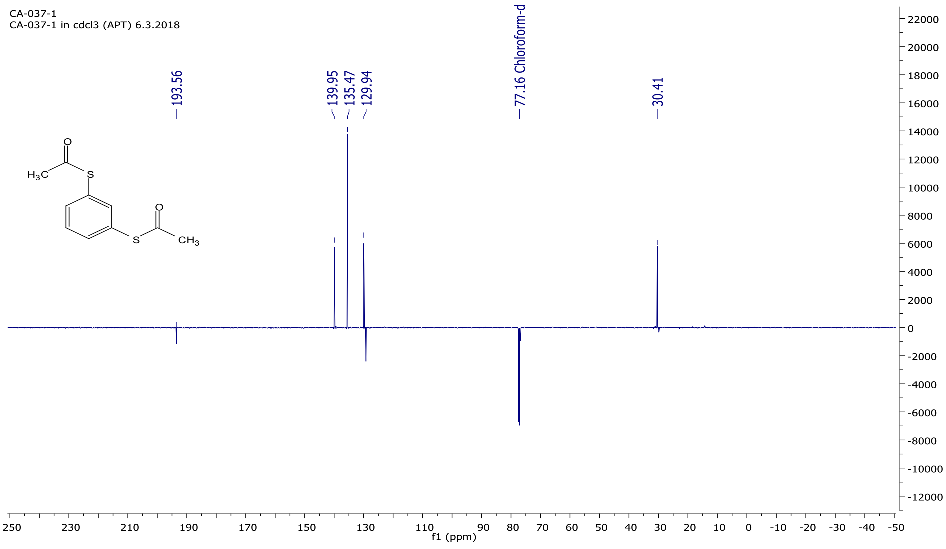

Figure S4 – ^{13}C NMR spectrum of **5**

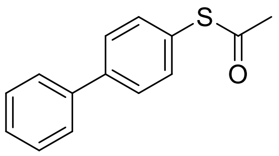

***S*-[1,1'-biphenyl]-4-yl ethanethioate (4b).** Pink solid; ^{1}H NMR (500 MHz, $CDCl_3$) δ (ppm) 7.65 – 7.62 (m, 2H, Ph H-3,5), 7.61 – 7.58 (m, 2H, Ph H-2',6'), 7.50 – 7.43 (m, 4H, Ph H-2,6,3',5'), 7.40 – 7.35 (m, 1H, Ph H-4'), 2.45 (s, 3H, CH_3); ^{13}C NMR (125 MHz, $CDCl_3$) δ (ppm) 194.22 (COS), 142.3 (Ph C-4), 140.14 (Ph C-1'), 134.76 (Ph C-2,6), 128.85 (Ph C-3',5'), 127.94 (Ph C-3,5), 127.78 (Ph C-4'), 127.20 (Ph C-2',6'), 126.69 (Ph C-1), 30,24 (CH_3); HRMS (ESI) *m/z*: calc. for $C_{14}H_{12}NaOS$: 251.0504 $[M+Na]^+$, found: 251.0501; R_f = 0.43 (*n*-hexane/EtOAc = 95:5); isolated yield: 90%

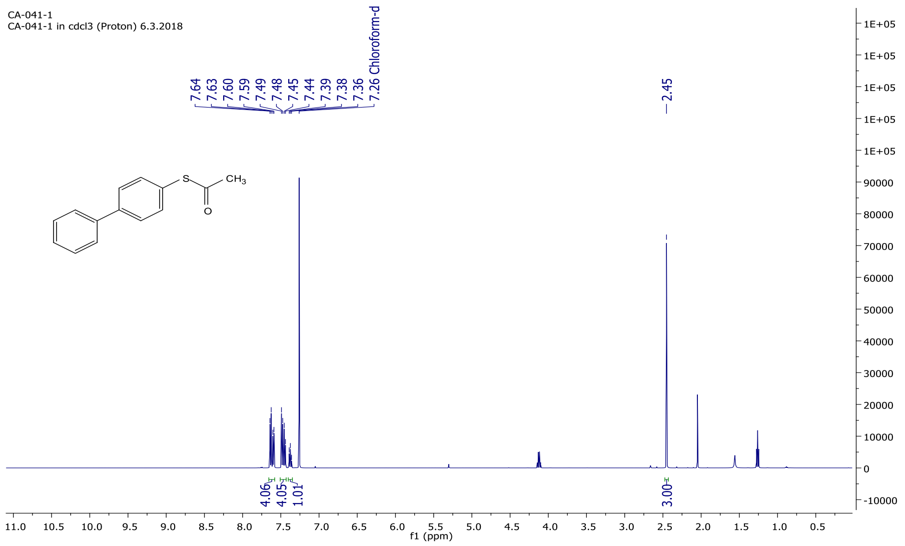

Figure S5 – ^{1}H-NMR spectrum of **4b**

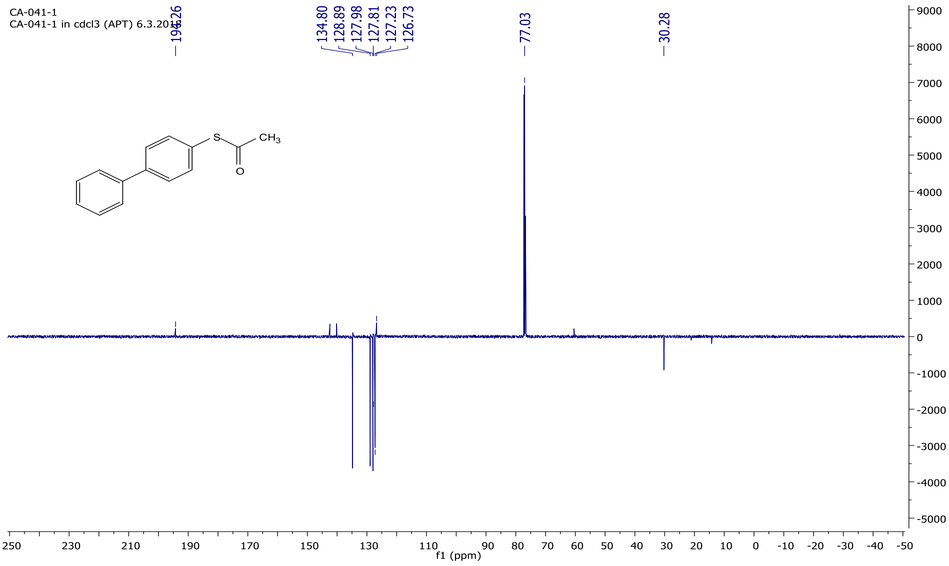

Figure S6 – ^{13}C NMR spectrum of **4b**

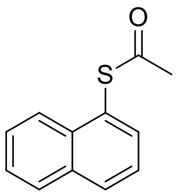

***S*-naphthalen-1-yl ethanethioate (4c).** Red oil; ^{1}H NMR (500 MHz, $CDCl_3$) δ (ppm) 8.20 (d, J = 8.3 Hz, 1H, Ar H-8), 7.99 (d, J = 8.3 Hz, 1H, Ar H-4), 7.89 (d, J = 7.8 Hz, 1H, Ar H-5), 7.71 (dd, J = 7.1, 1.2 Hz, 1H, Ar H-2), 7.57 – 7.51 (m, 3H, Ar H-7,6,3), 2.48 (s, 3H, CH_3); ^{13}C NMR (125 MHz, $CDCl_3$) δ (ppm) 194.11 (COS), 134.97 (Ar C-2), 134.13 (Ar C-4a, 8a), 130.97 (Ar C-4), 128.64 (Ar C-5), 127.19 (Ar C-7), 126.43 (Ar C-6); 125.57 (Ar C-3); 125.42 (Ar C-1), 125.23 (Ar C-8), 30.27 (CH_3); HRMS (ESI) *m/z*: calc. for $C_{12}H_{10}NaOS$: 225.0348 $[M+Na]^+$, found: 225.0345; R_f = 0.41 (*n*-hexane/EtOAc = 9:1); isolated yield: 71%

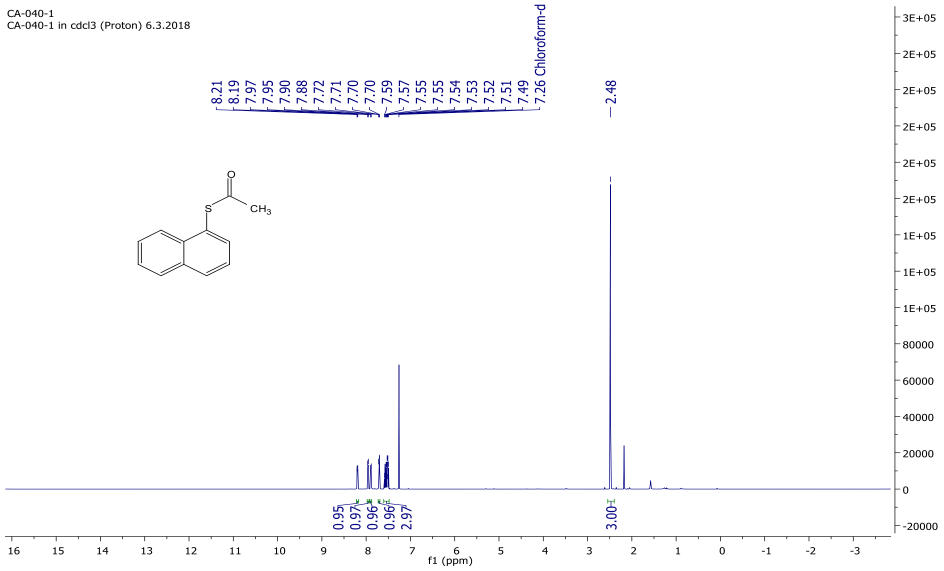

Figure S7 – ^{1}H-NMR spectrum of **4c**

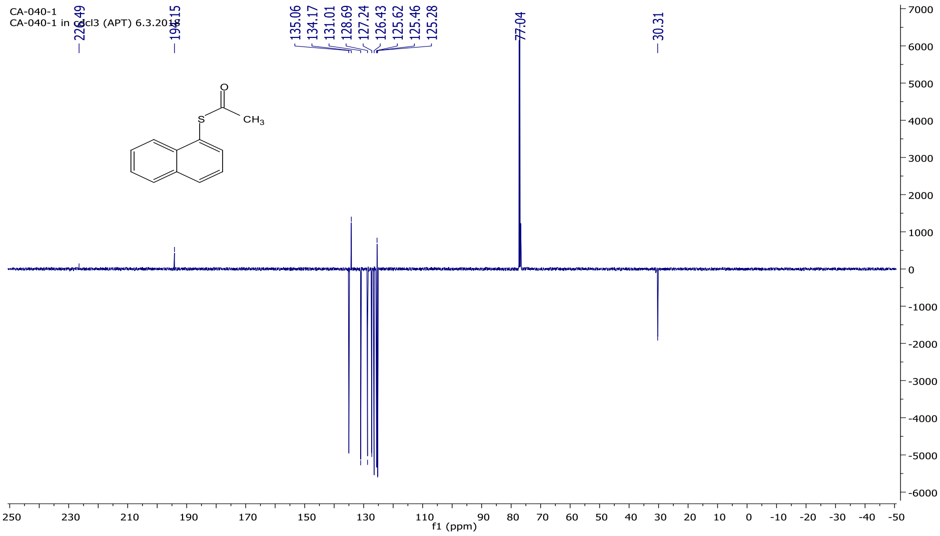

Figure S8 – ^{13}C NMR spectrum of **4c**

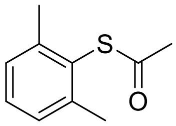

***S*-(2,6-dimethylphenyl) ethanethioate (4d).** Red oil; ^{1}H NMR (500 MHz, $CDCl_3$) δ (ppm) 7.24 - 721 (m, 1H, Ph H-4), 7.16 -7.14 (m, 2H, Ph H-3,5), 2.43 (s, 3H, $SCOCH_3$), 2.36 (s, 6H, Ar-CH_3); ^{13}C NMR (125 MHz, $CDCl_3$) δ (ppm) 193.37 (COS), 142.67 (Ph C-2,6), 129.90 (Ph C-4), 128.26 (Ph C-3,5), 127.27 (Ph C-1), 30.28 ($SCOCH_3$), 21.69 (Ar-CH_3); HRMS (ESI) *m/z*: calc. for $C_{10}H_{12}NaOS$: 203.0506 $[M+Na]^+$, found: 203.0501; R_f=0.43 (*n*-hexane/EtOAc = 9:1) isolated yield: 36%

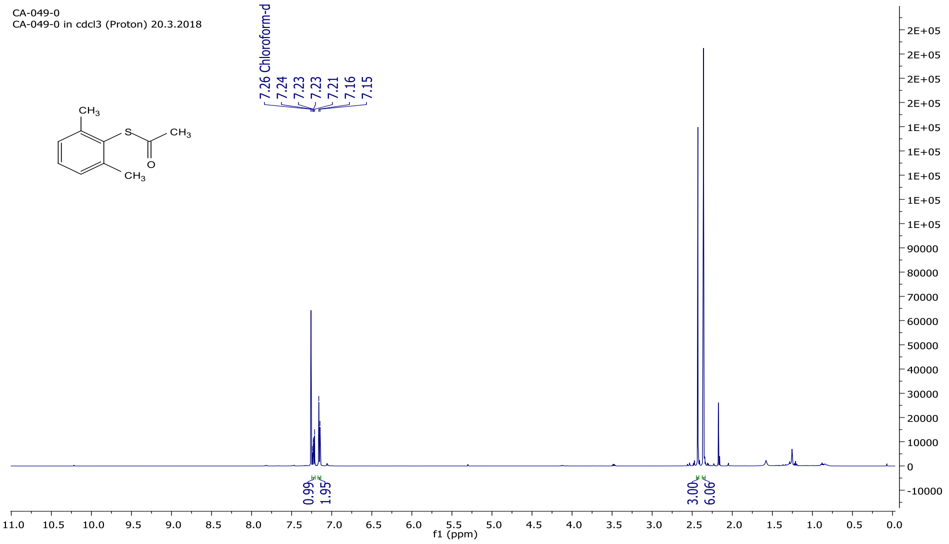

Figure S9 – ^{1}H NMR spectrum of **4d.**

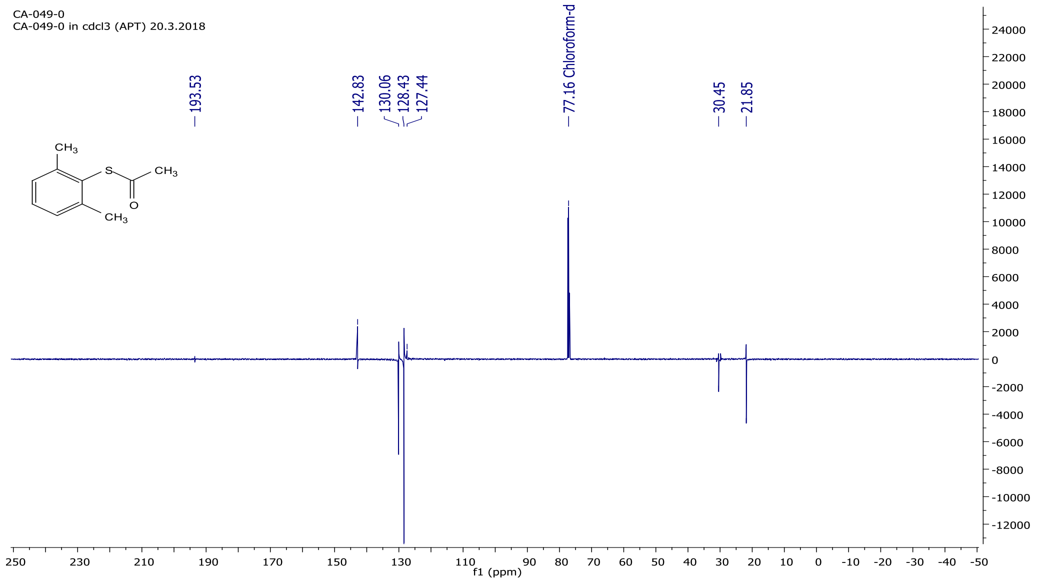

Figure S10 – ^{13}C NMR spectrum of **4d.**

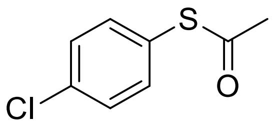

***S*-(4-chlorophenyl) ethanethioate (4e).** Brown oil; ^{1}H NMR (500 MHz, $CDCl_3$) δ (ppm) 7.40 - 7.38 (m, 2H, Ph H-3,5), 7.34 - 7.32 (m, 2H, Ph H-2,6), 2.43 (s, 3H, CH_3); ^{13}C NMR (125 MHz, $CDCl_3$) δ (ppm) 193.47 (COS), 135.84 (Ph C-4), 135.65 (Ph C-2,6), 129.45 (Ph C-3,5), 126.28 (Ph C-1), 30.19 (CH_3); HRMS (ESI) *m/z*: calc. for $C_8H_7ClNaOS$: 208.9801 $[M+Na]^+$, found: 208.9798; R_f = 0.35 (*n*-hexane/EtOAc = 9:1); isolated yield: 94 %

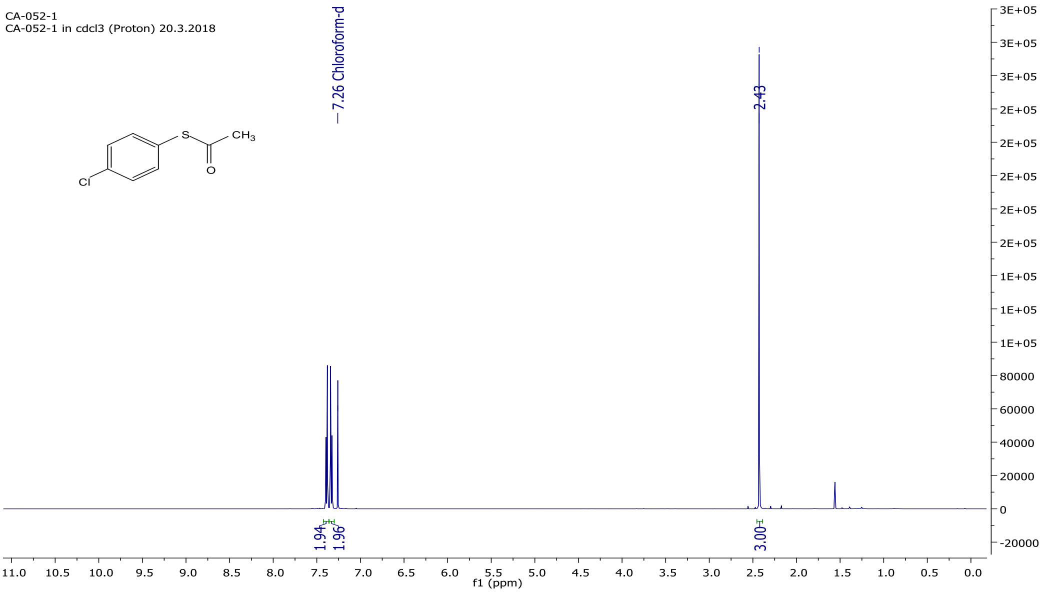

Figure S11 – ^{1}H NMR spectrum of **4e**

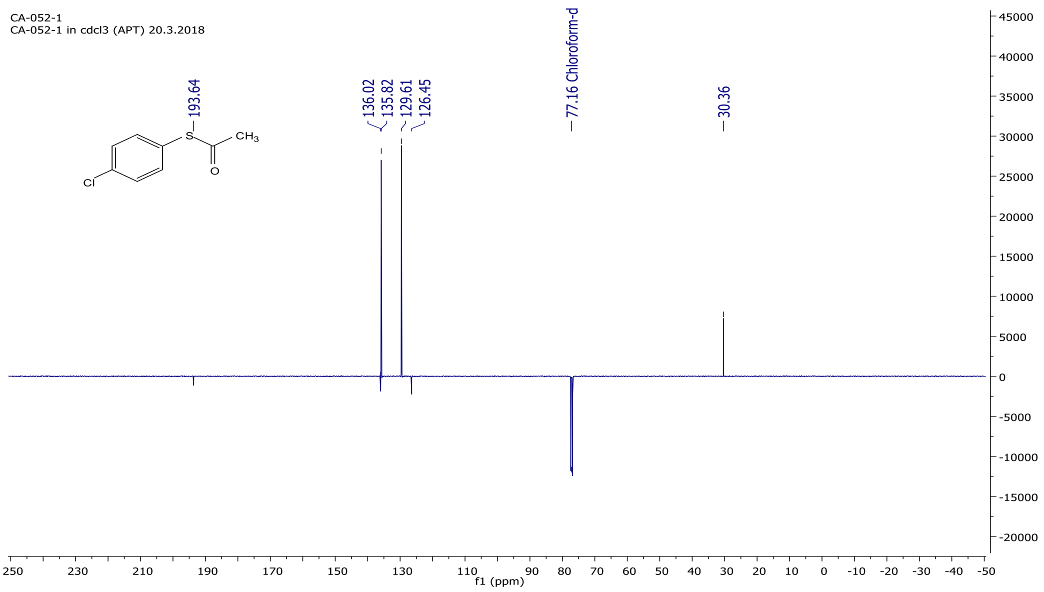

Figure S12 – ^{13}C NMR spectrum of **4e**

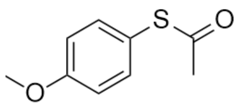

S-(4-methoxyphenyl) ethanethioate (4f). White solid. Mp: 98–101 °C. IR (KBr, cm^{-1}): ν 870, 1045, 1189, 1256, 1601, 1766. ^{1}H NMR (400 MHz, $CDCl_3$): δ 2.39 (s, 3H), 3.82 (s, 3H), 6.93 (d, J = 9 Hz, 2H), 7.31 (d, J = 9 Hz, 2H). ^{13}C NMR (100 Hz, $CDCl_3$): δ 30.1, 55.3, 115.0, 125.7, 135.7, 159.7, 194.5. HRMS (ESI) m/z: calc. for $C_9H_{10}NaO_2S$: 205.0299 $[M+Na]^+$, found: 205.0302; R_f = 0.32 (n-hexane/EtOAc = 9:1); isolated yield: 87%

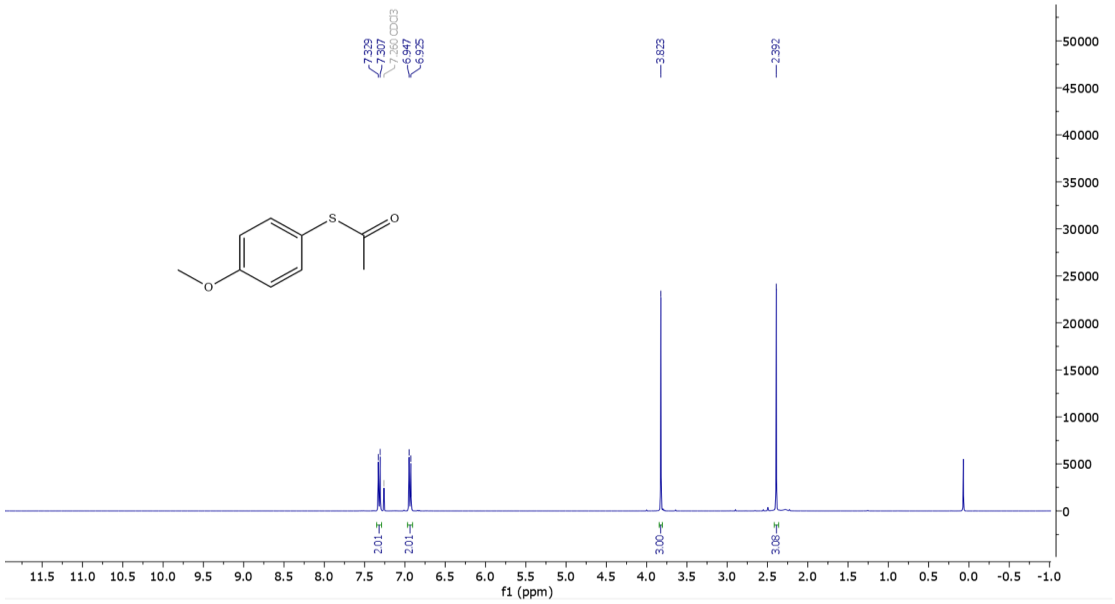

Figure S13 – ^{1}H NMR spectrum of **4f**

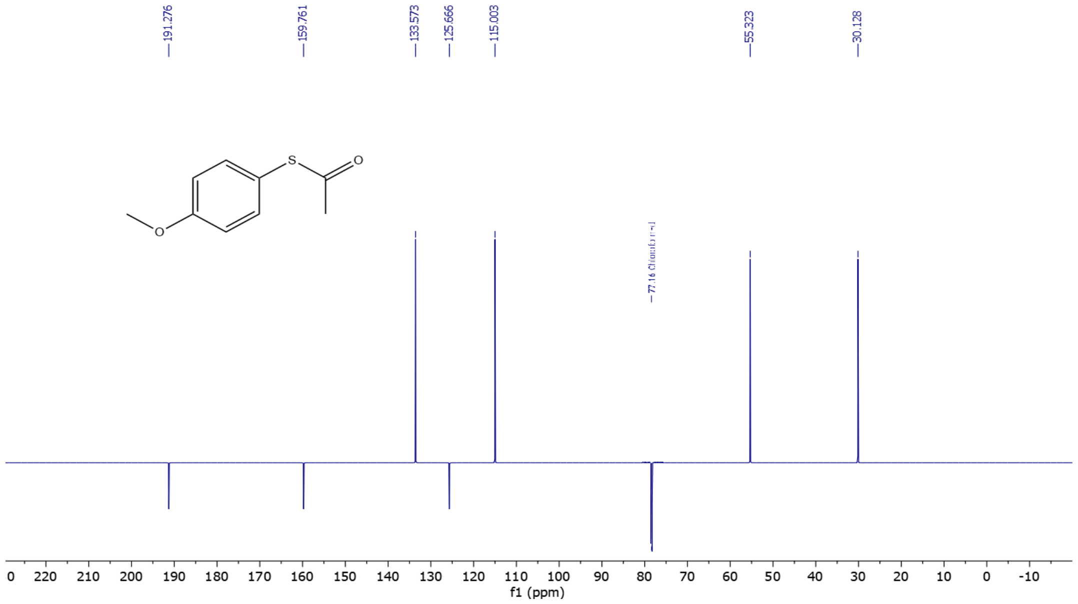

Figure S14 – ^{13}C NMR spectrum of **4f**

Flash chromatography purification of the reaction mixture obtained from compound **1g** gave derivatives **4g** and **6g** as a mixture in a similar ratio to that obtained in the reaction mixture (4g/6g = 90:10) (Figure 15)

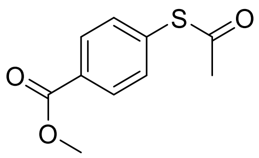

Methyl 4-(acetylthio)benzoate[1] (4g.^{1}HNMR (400 MHz, $CDCl_3$) δ 8.06 (d, *J* = 8.4 Hz, 2H), 7.50 (d, *J* = 8.4 Hz, 2H), 3.928 (s, 3H, OCH_3), 2.45 (s, 3H, $COCH_3$).

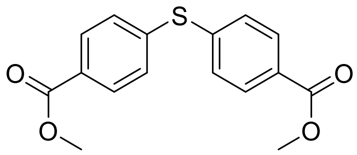

Bis(4-methoxyacetylphenyl)sulfane (6g)[2]**.** ^{1}HNMR (400 MHz, $CDCl_3$) δ 7.97 (d, *J* = 8.4 Hz, 2H), 7.38 (d, *J* = 8.4 Hz, 2H), 3.916 (s, 3H, OCH_3).

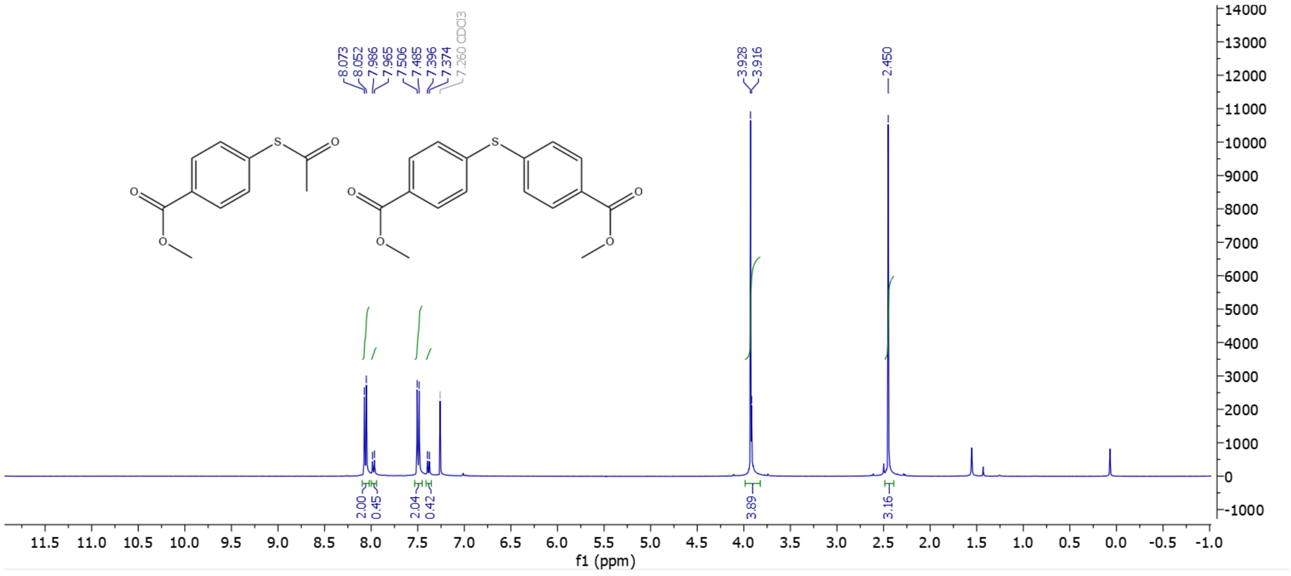

Figure S15 – ^{1}H NMR spectrum of **4g and 6g**

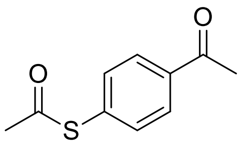

***S*-(4-acetylphenyl) ethanethioate (4h).** Red oil; ^{1}H NMR (500 MHz, $CDCl_3$) δ (ppm) 8.00 – 7.95 (m, 2H, Ph H-2,6), 7.55 – 7.49 (m, 2H, Ph H-3,5), 2.62 (s, 3H, $COCH_3$), 2.46 (s, 3H, $SCOCH_3$); ^{13}C NMR (125 MHz, $CDCl_3$) δ (ppm) 197.35 ($\underline{C}OCH_3$), 192.69 ($S\underline{C}OCH_3$), 137.34 (Ph C-4), 134.21 (Ph C-2,6), 133.73 (Ph C-1), 128.82 (Ph C-3,5), 30.46 ($CO\underline{C}H_3$), 26.69 ($SCO\underline{C}H_3$); HRMS (ESI) *m/z*: calc. for $C_{10}H_{10}NaO_2S$: 217.0297 $[M+Na]^+$, found: 217.0294; R_f = 0.44 (*n*-hexane/EtOAc = 95:5); isolated yield: 87%

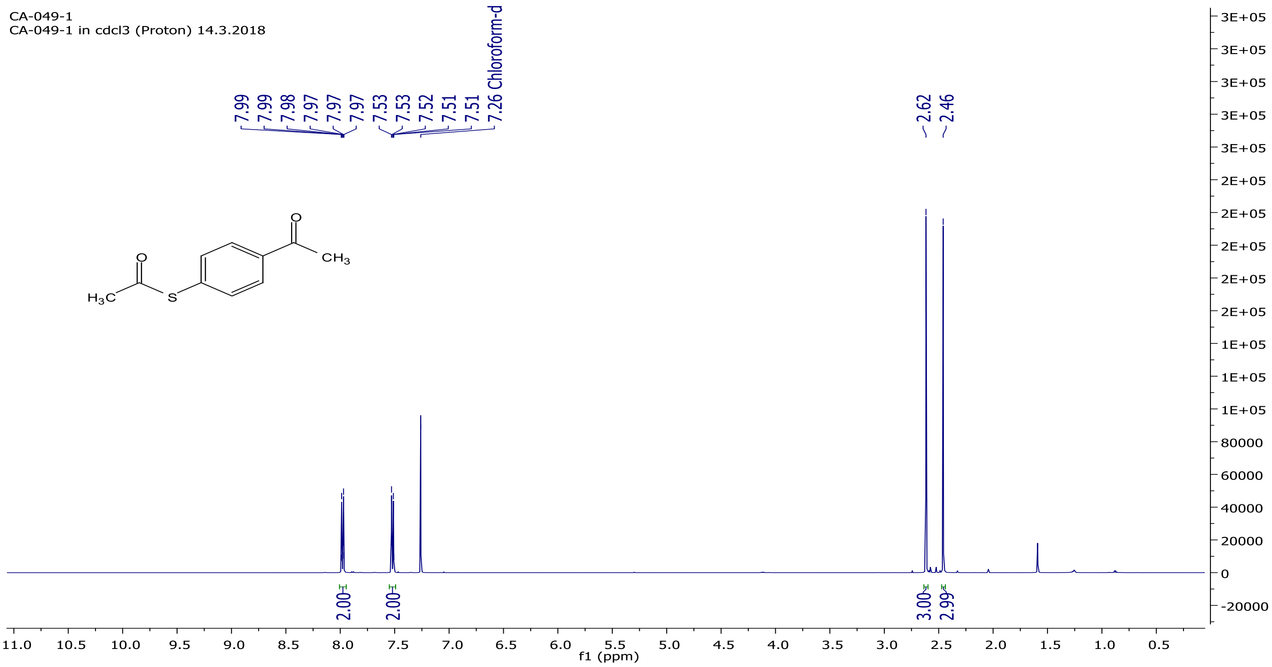

Figure S16 – ^{1}H-NMR spectrum of **4h**.

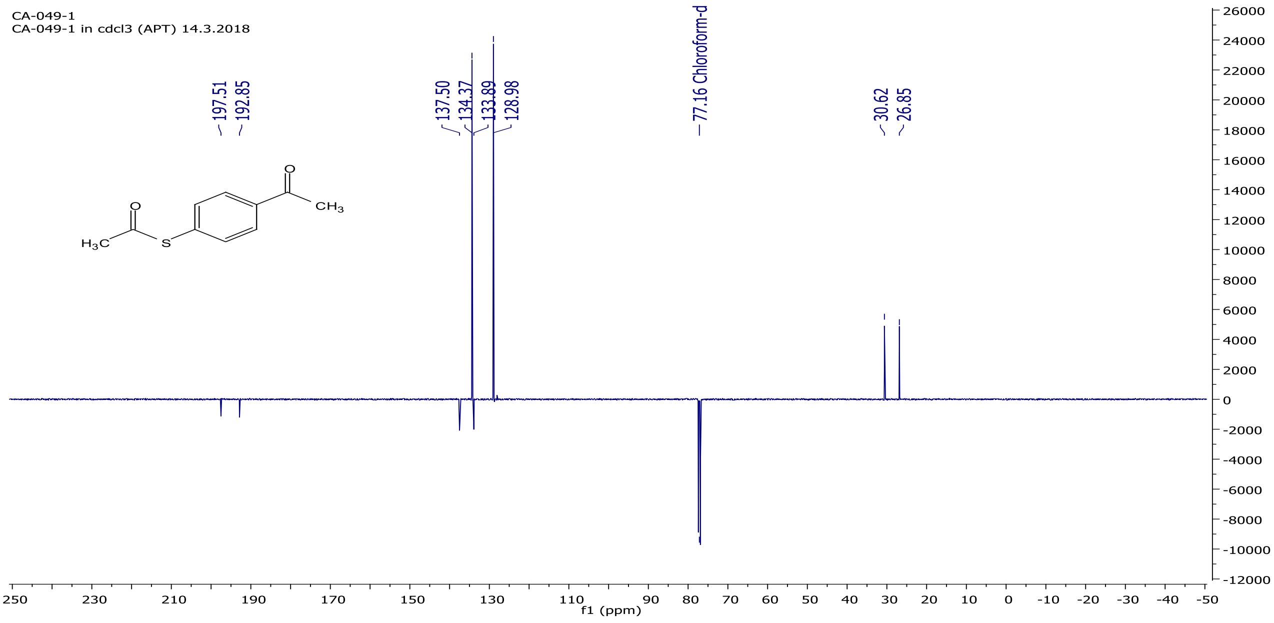

Figure S17 – ^{13}C NMR spectrum of **4h**.

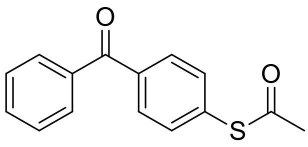

***S*-(4-benzoylphenyl) ethanethioate (4i).** Yellow oil; ^{1}H NMR (500 MHz, $CDCl_3$) δ (ppm) 7.84 – 7.80 (m, 4H, Ph H-2',6',3,5), 7.62 - 7.58 (m, 1H, Ph H-4'), 7.55 – 7.53 (m, 2H, Ph H-2,6), 7.51 – 7.48 (m, 2H, Ph H-3',5'), 2.47 (s, 3H, $SCOCH_3$); ^{13}C NMR (125 MHz, $CDCl_3$) δ (ppm) 195.87 (PhCOPh), 192.78 ($S\underline{C}OCH_3$), 138.01 (Ph C-4), 137.12 (Ph C-1'), 133.88 (Ph C-2,6), 132.94 (Ph C-1), 132.69 (Ph C-4'), 130.51 (Ph C-3,5), 130.04 (Ph C-2',6'), 128.37 (Ph C-3',5'), 30.45 ($SCO\underline{C}H_3$); HRMS (ESI) *m/z*: calc. for $C_{15}H_{12}NaO_2S$: 279.0457 $[M+Na]^+$, found: 279.0450; R_f = 0.39 (*n*-hexane/EtOAc = 95:5) isolated yield: 87%

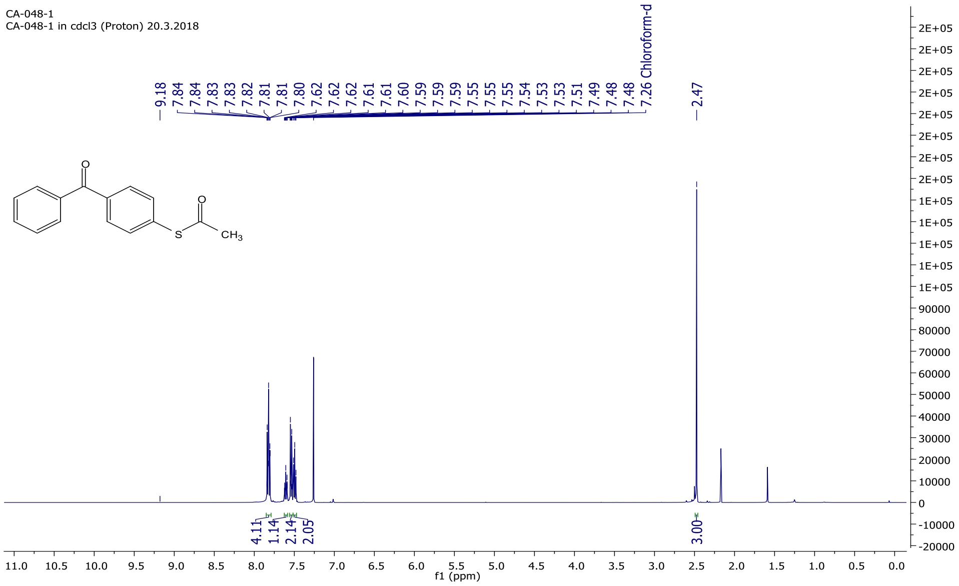

Figure S18 – ^{1}H-NMR spectrum of **4i**.

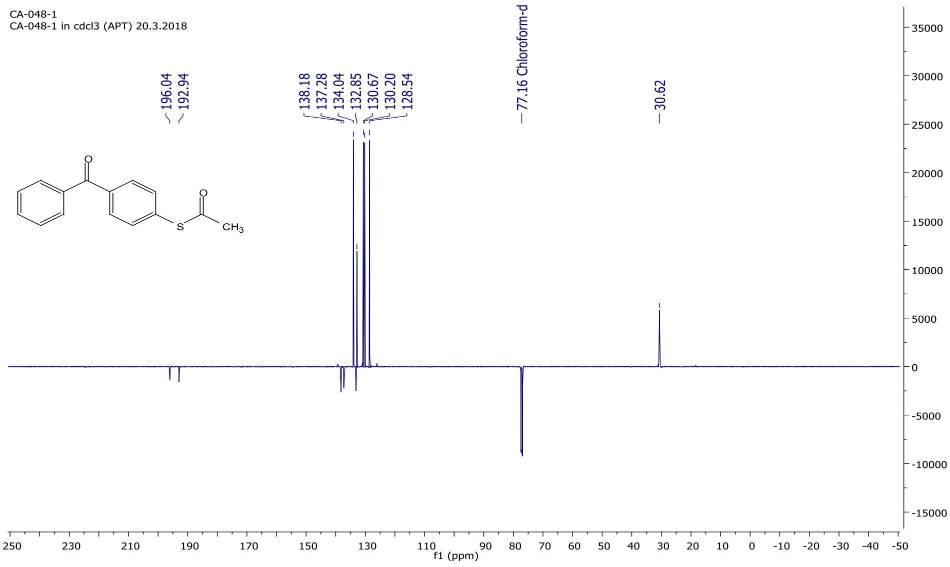

Figure S19– ^{13}C NMR spectrum of **4i**.

Flash chromatography purification of the reaction mixture, obtained from compound **1j,** gave derivatives **4j** and **6j** as a mixture in a similar ratio to that obtained in the reaction mixture (4j/6j = 70:30) (Figure 15)

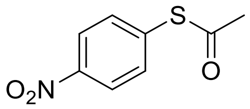

***S*-(4-nitrophenyl) ethanethioate (4j).**[3] 1H NMR (200 MHz, $CDCl_3$) δ 8.25 (d, *J* = 8.0 Hz, 2H), 7.60 (d, *J* = 8.9 Hz, 2H), 2.49 (s, 3H, CH_3).

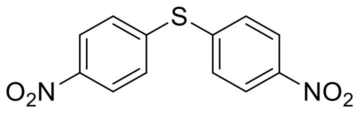

Bis(4-nitrophenyl)sulfane (6j)[4] 1H NMR (200 MHz, $CDCl_3$) δ 8.18 (d, *J* = 8.8 Hz, 4H), 7.49 (d, *J* = 8.9 Hz, 4H).

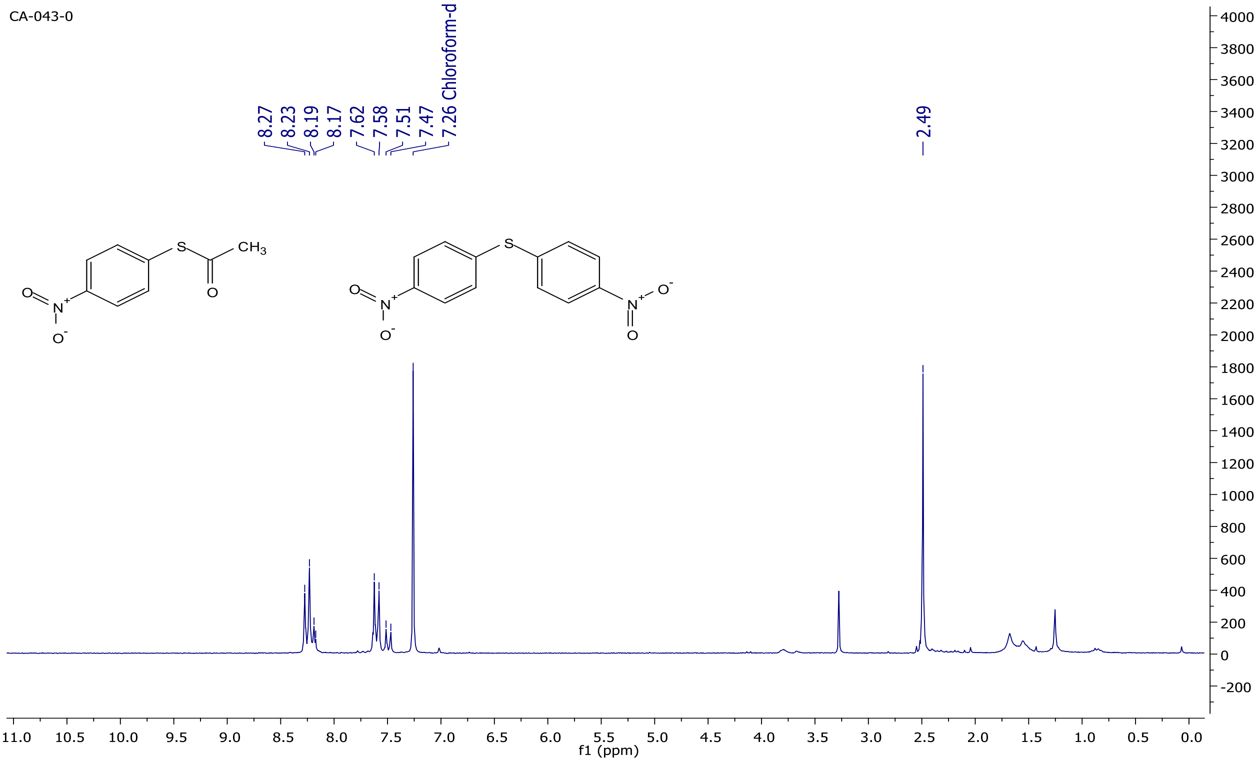

Figure S20 – ^{1}H-NMR spectrum of the **11c** and **11d** mixture.

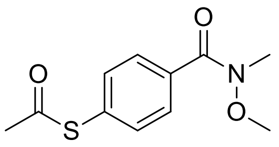

***S*-(4-(methoxy(methyl)carbamoyl)phenyl) ethanethioate (4k).** Brown oil; ^{1}H NMR (500 MHz, $CDCl_3$) *δ* (ppm) 7.72 – 7.70 (m, 2H, Ph H-3,5), 7.46 – 7.44 (m, 2H, Ph H-2,6), 3.55 (s, 3H, OCH_3), 3.36 (s, 3H, $CONCH_3$), 2.43 (s, 3H, $SCOCH_3$); ^{13}C NMR (125 MHz, $CDCl_3$) *δ* (ppm) 193.07 (COS), 168.85 (CON), 134.72 (Ph C-4), 133.66 (Ph C-2,6), 130.74 (Ph C-1), 128.89 (Ph C-3,5), 61.16 (OCH_3), 33.57 (NCH_3), 30.31 (SCO$\underline{C}H_3$); HRMS (ESI) *m/z*: calc. for $C_{11}H_{13}NNaO_3S$: 262.0511 $[M+Na]^+$, found: 262.0508; R_f = 0.28 (*n*-hexane/EtOAc = 6:4); isolated yield: 76%

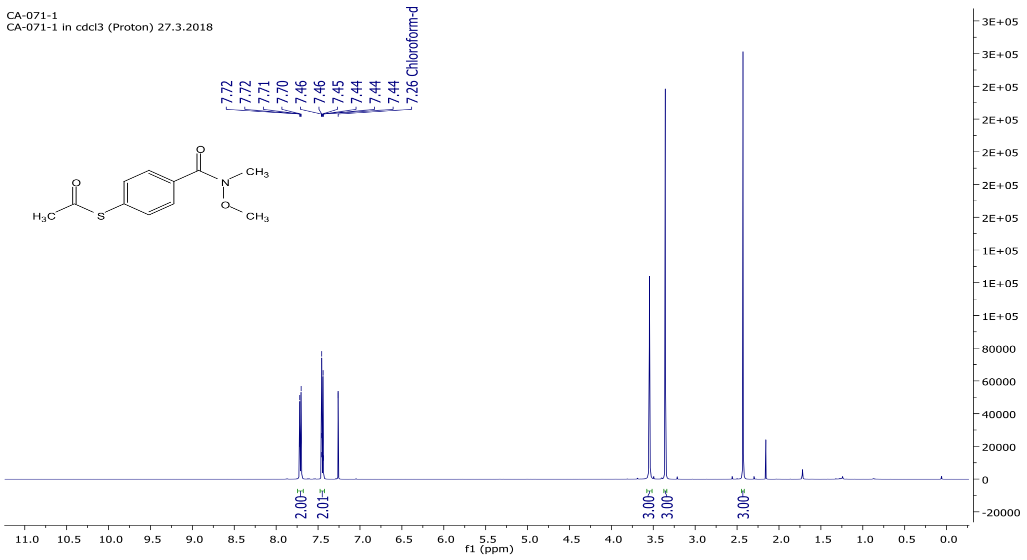

Figure S21 – ^{1}H-NMR spectrum of **4k**.

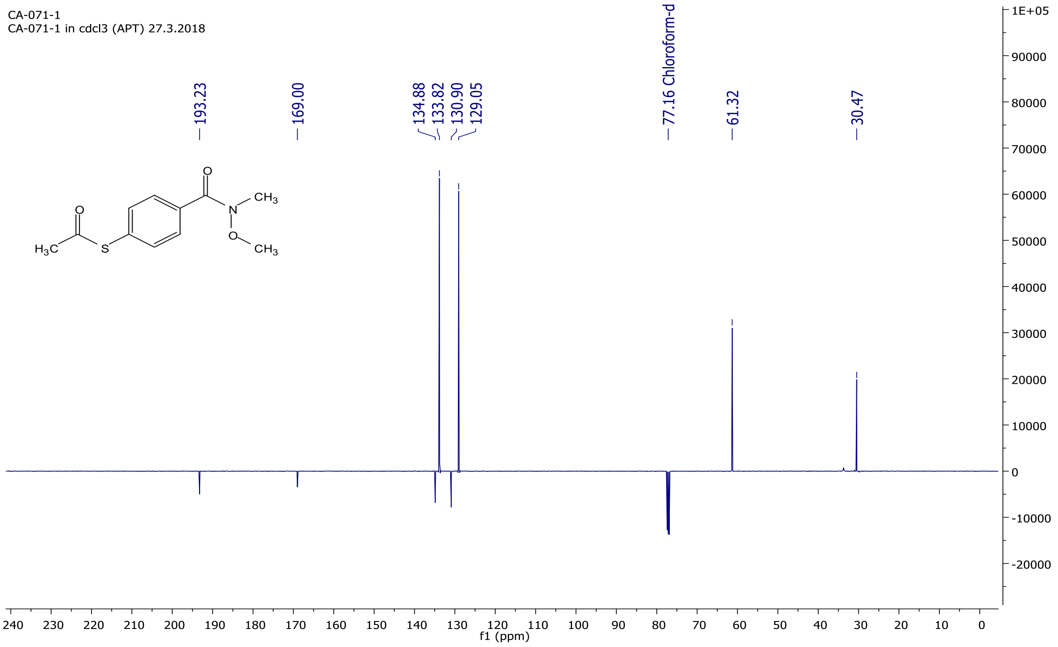

Figure S22 – ^{13}C NMR spectrum of **4k**.

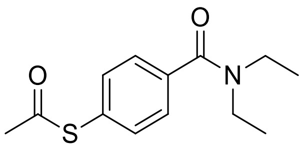

***S*-(4-(diethylcarbamoyl)phenyl) ethanethioate (4l).** Brown oil; ^{1}H NMR (500 MHz, $CDCl_3$) *δ* (ppm) 7.45 - 7.43 (m, 2H, Ph H-2,6), 7.41 – 7.39 (m, 2H, Ph H-3,5), 3.54 (bs, 2H, CH_2), 3.26 (bs, 2H, CH_2), 2.43 (s, 3H, $SCOCH_3$), 1.25 (bs, 3H, CH_3), 1.12 (bs, CH_3); ^{13}C NMR (125 MHz, $CDCl_3$) *δ* (ppm) 193.37 (COS), 170.28 (CON), 138.09 (Ph C-4), 134.24 (Ph C-2,6), 129.07 (Ph C-1), 127.06 (Ph C-3,5), 43.27 (CH_2), 39.25 (CH_2), 30.28 (SCO$\underline{C}H_3$), 14.22 (CH_3), 12.84 (CH_3); HRMS (ESI) *m/z*: calc. for $C_{13}H_{17}NNaO_2S$: 274.0876 $[M+Na]^+$, found: 274.0872; R_f = 0.166 (*n*-hexane/EtOAc = 6:4); isolated yield: 62%

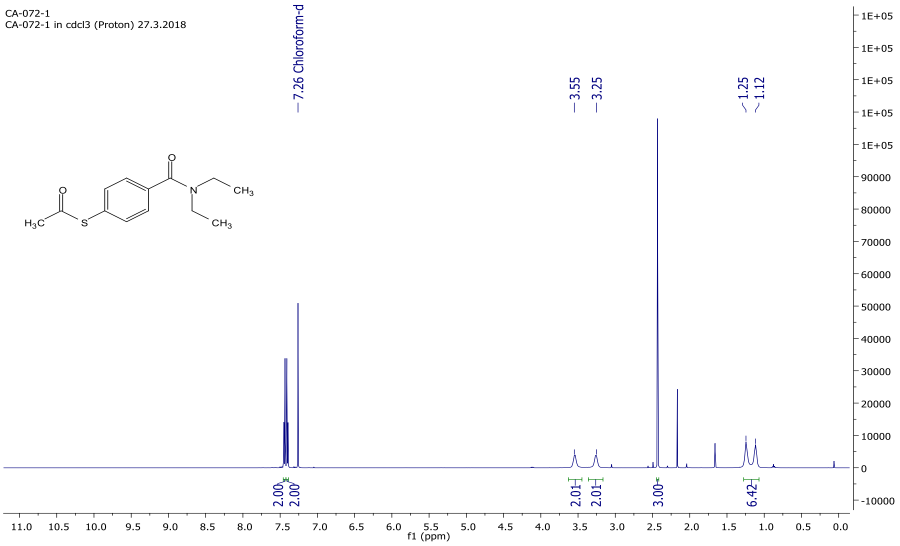

Figure S23 – ^{1}H-NMR spectrum of **4l**.

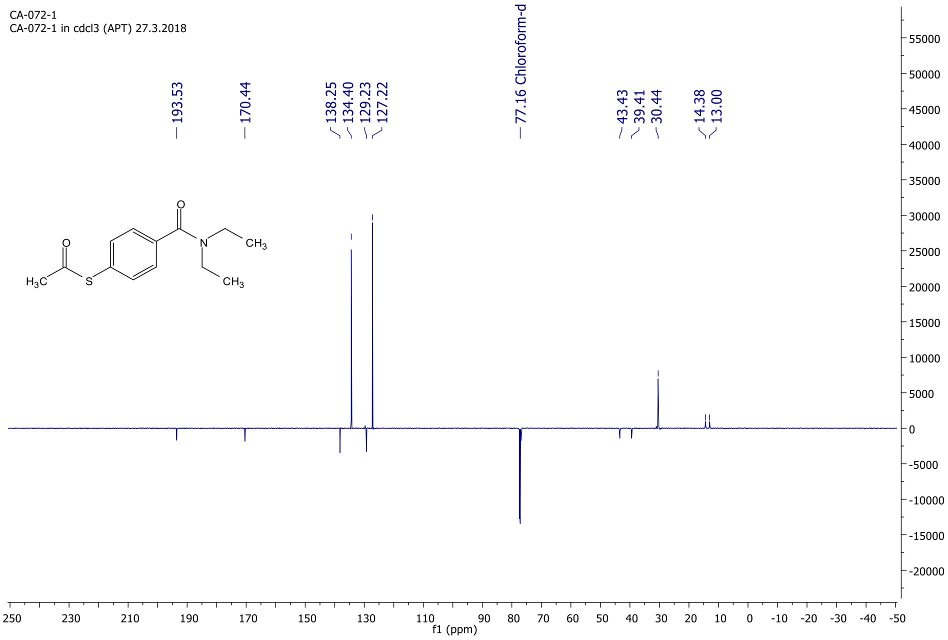

Figure S24 – ^{13}C NMR spectrum of **4l**.

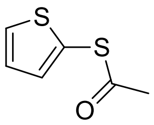

***S*-thiophen-2-yl ethanethioate (4m).** Red oil; ^{1}H NMR (500 MHz, $CDCl_3$) δ (ppm) 7.56 (dd, J = 5.3, 1.3 Hz, 1H, Th H-5), 7.17 (dd, J = 3.6, 1.3 Hz, 1H, Th H-3), 7.11 (dd, J = 5.3, 3.6 Hz, 1H, Th H-4), 2.41 (s, 3H, CH_3); ^{13}C NMR (125 MHz, $CDCl_3$) δ (ppm) 194.29 (COS), 135.80 (Th C-3), 131.91 (Th C-5), 127.88 (Th C-4), 125.00 (Th C-2), 29.49 (CH_3); HRMS (ESI) *m/z*: calc. for $C_6H_6NaOS_2$: 180.9751 $[M+Na]^+$, found: 180.9752; R_f = 0.44 (*n*-hexane/EtOAc = 95:5); isolated yield: 90%

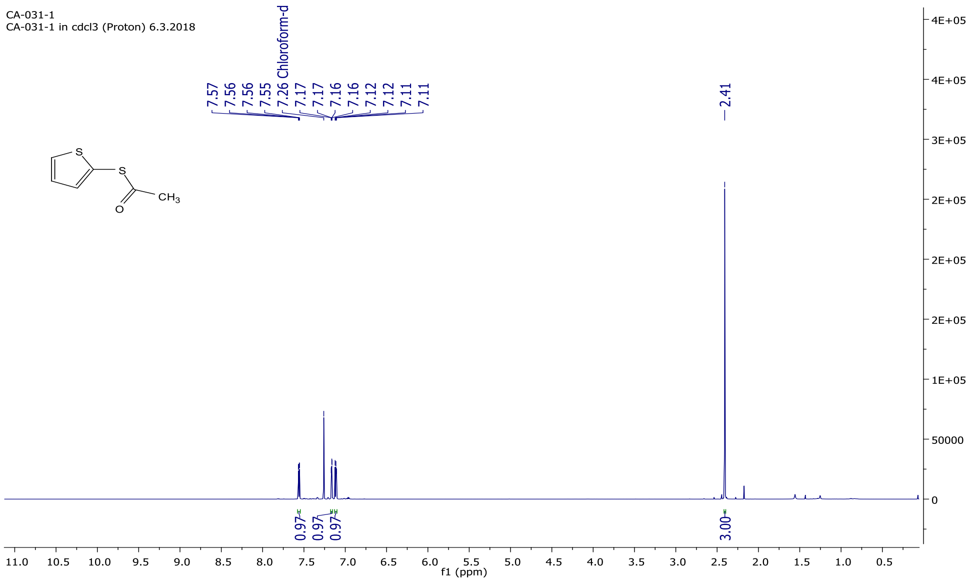

Figure S25 – ^{1}H-NMR spectrum of **4m**.

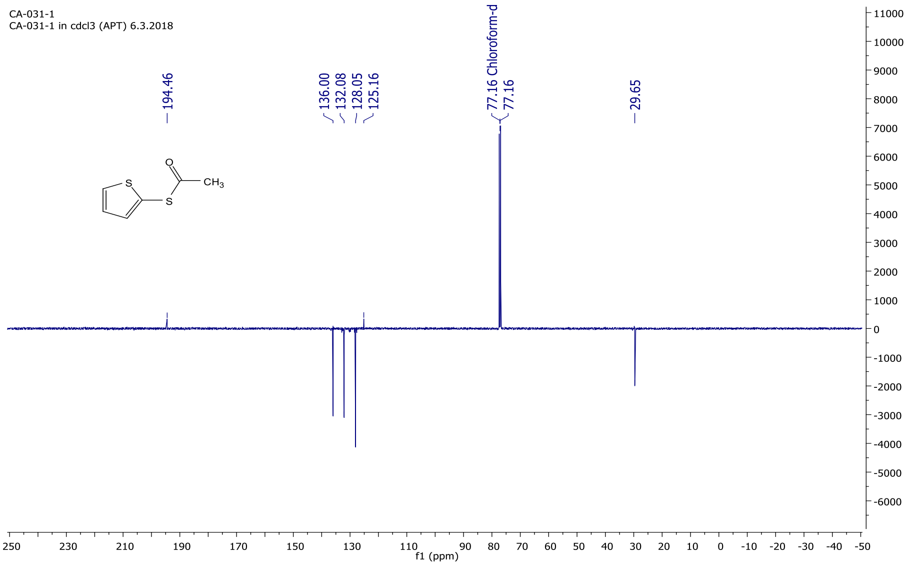

Figure S26 – ^{13}C NMR spectrum of **4m**.

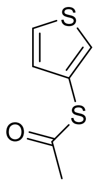

***S*-thiophen-3-yl ethanethioate (4n).** Pale red oil; ^{1}H NMR (500 MHz, $CDCl_3$) δ (ppm)7.46 (dd, J = 3.0, 1.2 Hz, 1H, Th H-2), 7.41 (dd, J = 5.0, 3.0 Hz, 1H, Th H-4), 7.07 (dd, J = 5.0, 1.2 Hz, 1H, Th H-5), 2.40 (s, 3H); ^{13}C NMR (125 MHz, $CDCl_3$) δ (ppm) 194.16 (COS), 131.32 (Th C-5), 130.19 (Th C-2), 126.32 (Th C-4), 123.27 (Th C-3), 29.91 (CH_3); HRMS (ESI) m/z: calc. for C6H6NaOS2: 180.9755 $[M+Na]^{+}$, found: 180.9752; R_f = 0.41 (n-hexane/EtOAc = 95:5); isolated yield: 72%

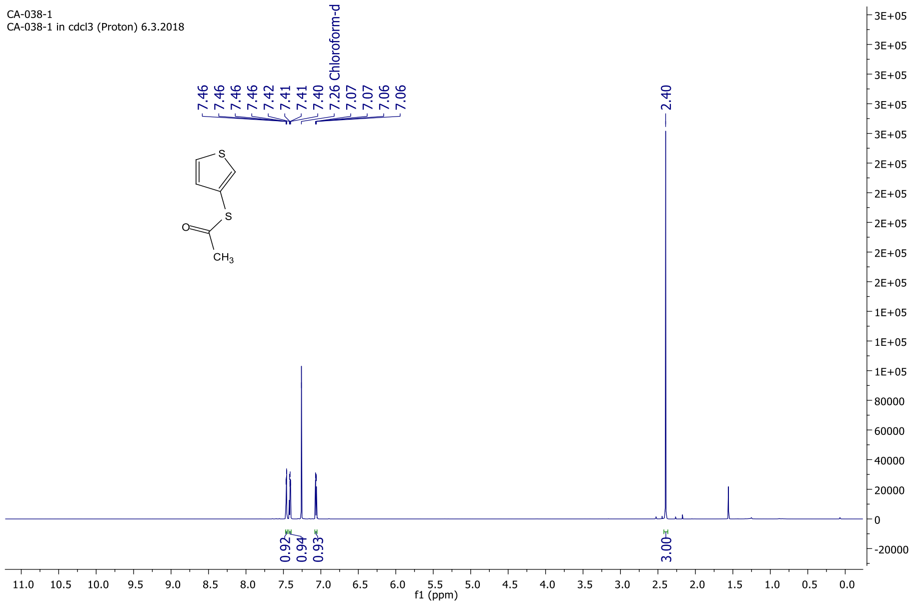

Figure S27 – ^{1}H-NMR spectrum of **4n**.

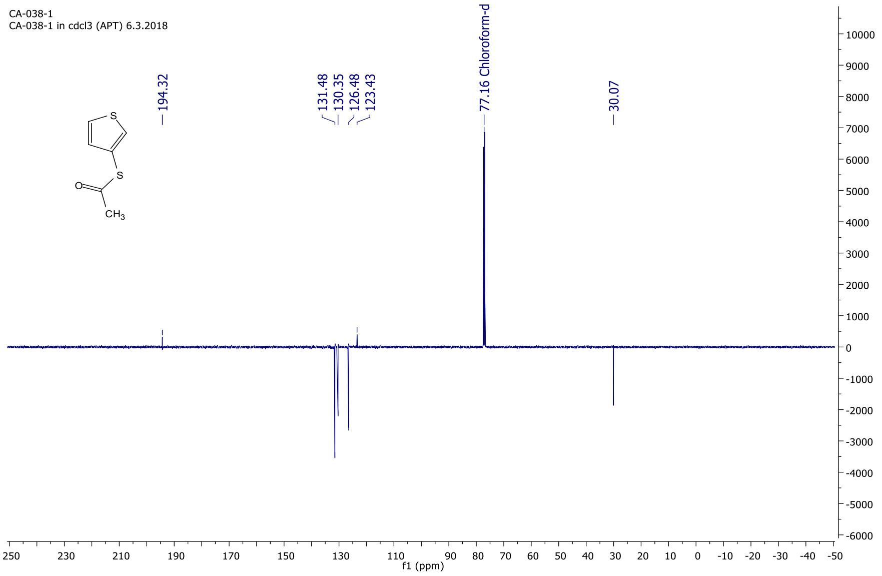

Figure S28 – ^{13}C NMR spectra of **4n**.

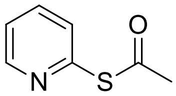

***S*-pyridin-2-yl ethanethioate (4o).** Yellow oil; ^{1}H NMR (500 MHz, $CDCl_3$) δ (ppm) 8.62 (ddd, J = 4.9, 2.0, 0.9 Hz, 1H, Py H-6), 7.74 (td, J = 7.7, 1.9 Hz, 1H, Py H-4), 7.61 (dt, J = 7.9, 1.0 Hz, 1H, Py H-3), 7.29 (ddd, J = 7.5, 4.8, 1.1 Hz, 1H, Py H-5), 2.46 (s, 3H); ^{13}C NMR (125 MHz, $CDCl_3$) δ (ppm) 193.16 (COS), 151.61 (Py C-2), 150.54 (Py C-6), 137.30 (Py C-4), 130.19 (Py C-3), 123.69 (Py C-5), 30.87 (Py CH_3); HRMS (ESI) m/z: calc. for C_7H_7NNaOS: 176.0136 $[M+Na]^+$, found: 176.0141; R_f = 0.213 (n-hexane/EtOAc = 8:2); isolated yield: 57%

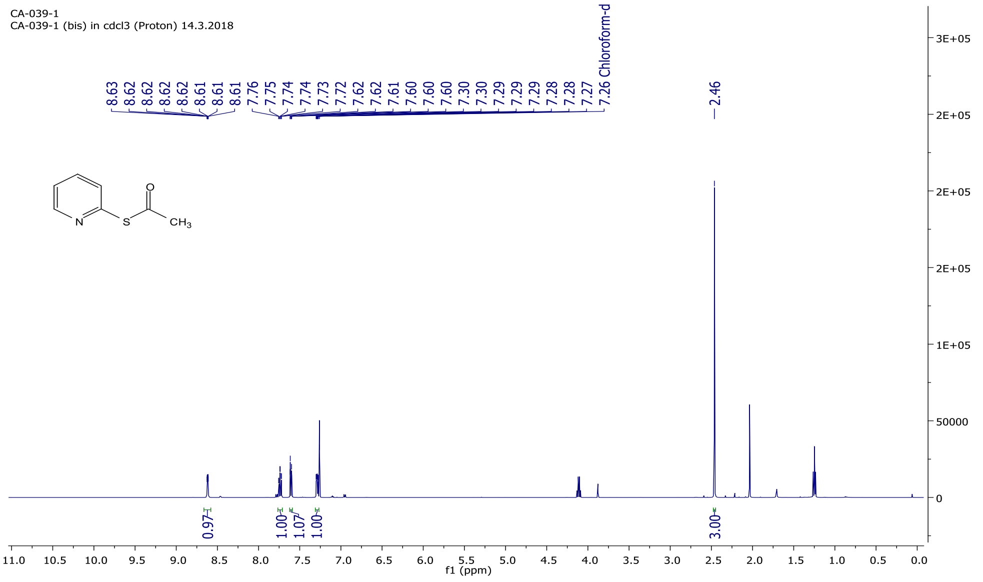

Figure S29 – ^{1}H-NMR spectra of **4o**.

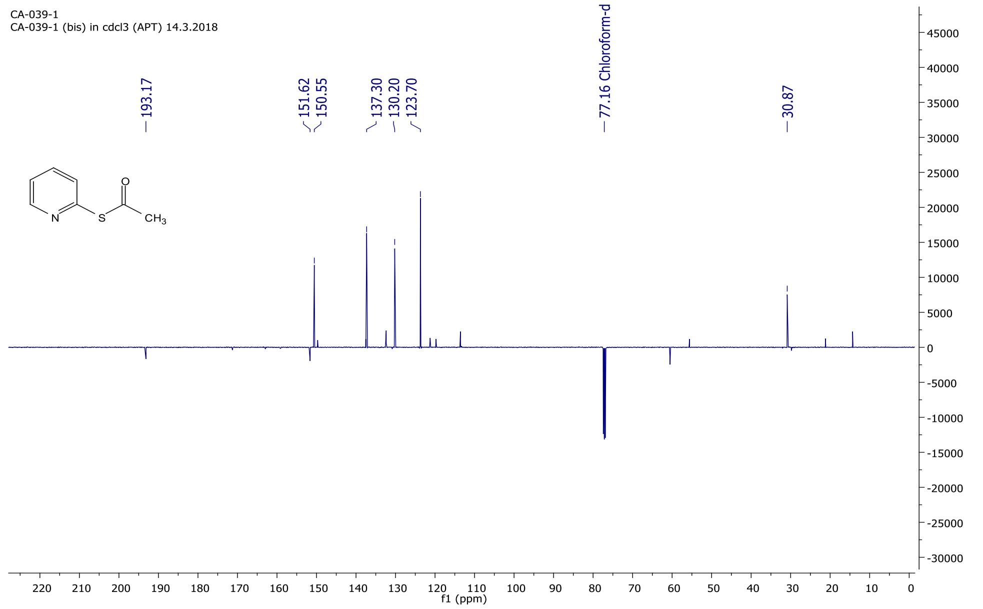

Figure S30 – ^{13}C NMR spectrum of **4o**.

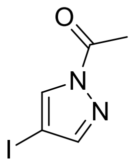

***N*-acetyl-4-iodopyrazole (9).** White crystals; Mp 112°C (AcOEt); ^{1}H NMR (500 MHz, $CDCl_3$) δ (ppm) 8.32 (s, 1H, Ar H-5), 7.68 (s, 1H, Ar H-4), 2.68 (s, 3H); ^{13}C NMR (125 MHz, $CDCl_3$) δ (ppm) 168.25 (CON), 148.43 (Ar C-3), 132.74 (Ar C-5), 63.56 (Ar C-4), 21.15 (CH_3); R_f = 0.293 (*n*-hexane/EtOAc = 8:2); isolated yield: 96%

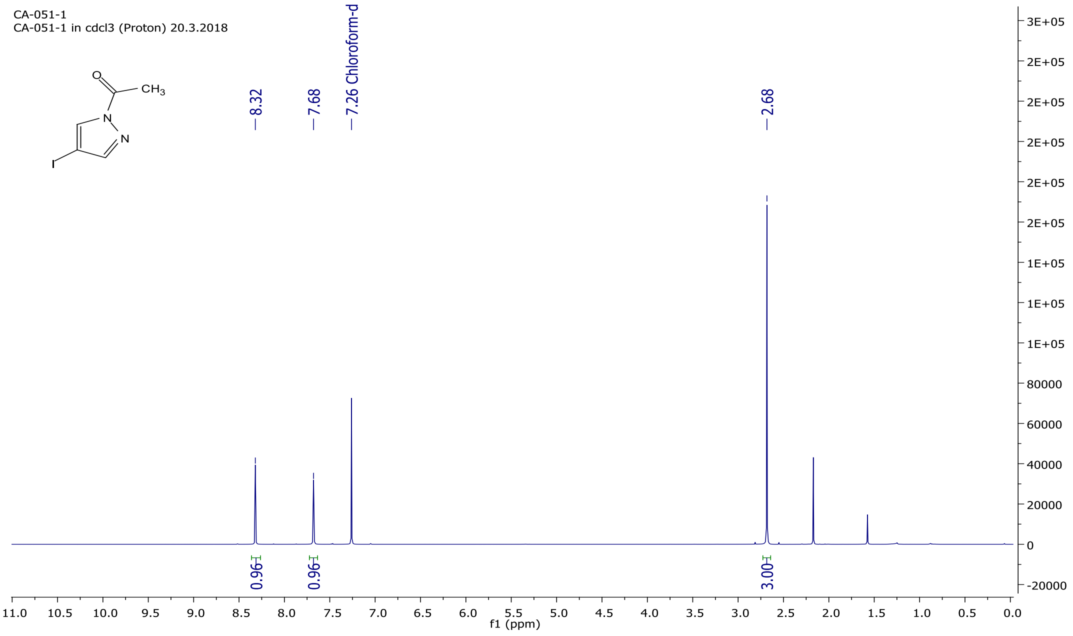

Figure S31 – ^{1}H-NMR spectrum of **9**.

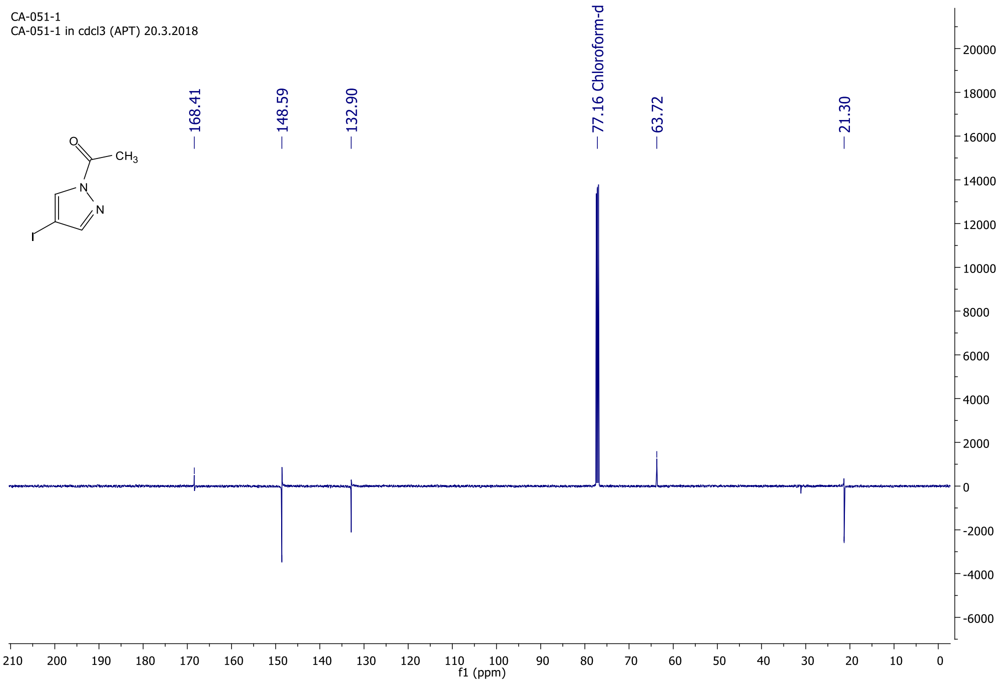

Figure S32 – ^{13}C NMR spectrum of **9**.

Synthesis of 1,2-Bis(6-iodo-2-methyl-1-benzothiophen-3-yl)hexafluorocyclopentene (10)[5]. A 250 ml Schlenk flask filled with 1,2-Bis(2-methylbenzo[b]thiophen-3-yl) hexafluorocyclopentene (2.88 g, 6.14 mmol), synthesized according to a literature procedure,[39] sulfuric acid (1.7 ml), water (5 ml), and AcOH (125 ml) was heated to 70 °C. Iodine (1.45 g, 5.71 mmol, 0.93 eq.) and H_5IO_6 (0.49 g, 2.15 mmol, 0.35 eq.) were added to the above clear solution and reacted for 3 h. A small amount of iodine was then added (0.6 mmoli; 0.16 g; 0.1 eq) and reacted for 5h at 70 °C. Then the mixture was poured into 150 ml of ice and filtered on a gooch funnel washing with water and a saturated solution of $Na_2S_2O_4$. The solid was solubilized in Et_2O (100 mL), dried with $MgSO_4$ and the solvent evaporated in vacuo. Purification by chromatography on silica gel (cyclohexane/AcOEt 9.5:0.5) give 5 in 49% yield (3.01 mmoli, 2.17 g) and a fraction with a mixture of 5 and the corresponding mono-iodo derivative in a ratio 7:3, which was reacted under the same conditions giving, after recrystallization with CH_3OH, 1.41 g of 5 (1.95 mmol) (overall yield 81%). The product consists of two conformational isomers parallel (10p) and antiparallel (10ap) as yet reported.[5]

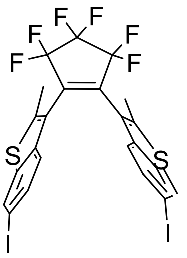

10p

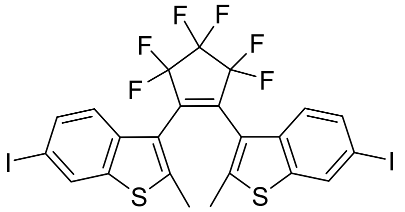

10ap

^{1}H NMR (400 MHz, $CDCl_3$) antiparallel conformer δ (ppm) 8.04 (s, 2H), 7.65 (d, J = 8.5 Hz, 2H) 7.36 (d, J = 8.6 Hz, 2H), 2.18 (s, 6H).); parallel conformer δ (ppm) 7.97 (s, 2H), 7.48 (d, J = 8.4 Hz, 2H), 7.22 (d, J = 8.6 Hz, 2H), 2.46 (s, 6H). Parallel/antiparallel = 37:63.

^{13}C NMR (100 MHz, $CDCl_3$) antiparallel conformer δ (ppm) 143.47, 140.23, 137,55, 133.77, 130.81, 123.48, 119.05, 89.20, 15.22 parallel conformer δ (ppm)143.11, 140.13, 137.55, 133.67, 130.66, 123.53, 123.16, 119.22, 89.35, 15.29.

^{1}H/^{13}C HSQC NMR ($CDCl_3$) antiparallel conformer δH/δC δ (ppm) 8.04/130.81, 7.65/133.70, 7.36/123.41, 2.45/15.25; parallel conformer δH/δC δ (ppm) 7.97/130.65, 7.48/133.72, 7.22/132.11, 2.17/15.25

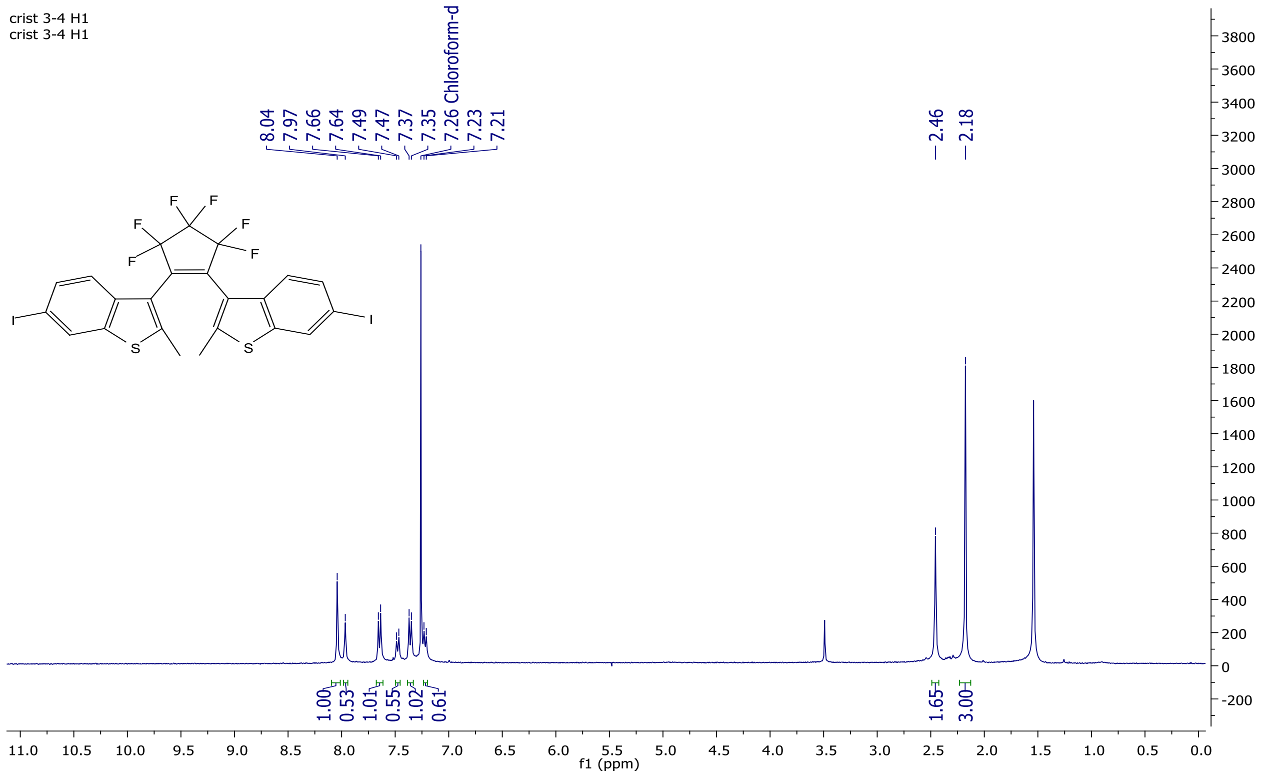

Figure S33 – 1,2-Bis(6-iodo-2-methyl-1-benzothiophen-3-yl)hexafluorocyclopentene (**10**) ^{1}H NMR spectrum

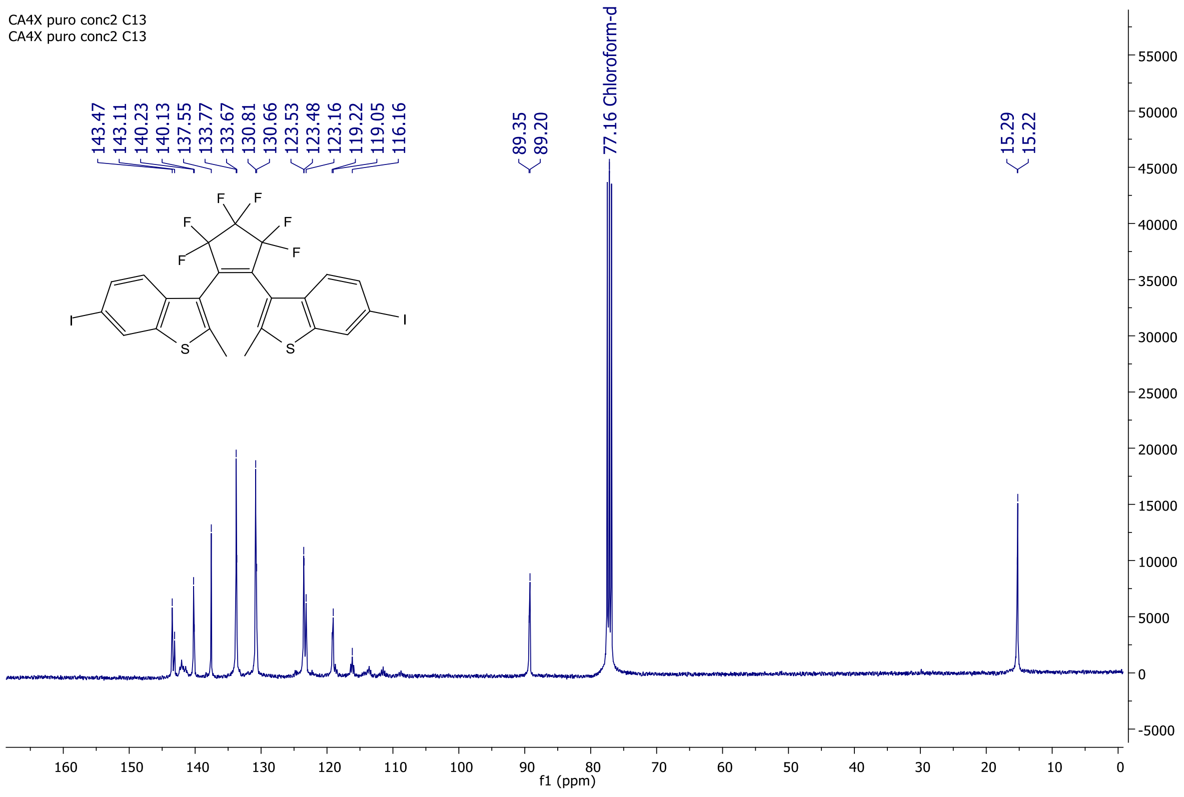

Figure S34 – 1,2-Bis(6-iodo-2-methyl-1-benzothiophen-3-yl)hexafluorocyclopentene (**10**) ^{13}C NMR spectrum

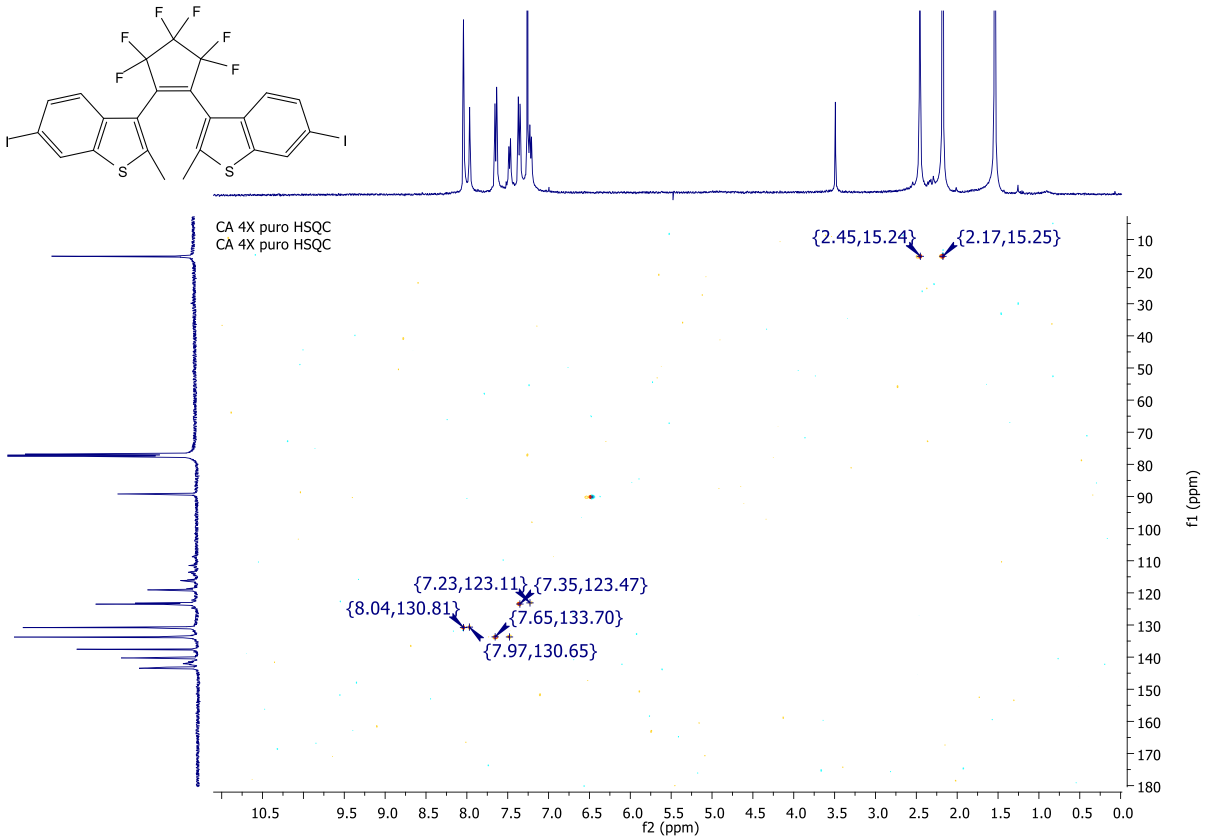

Figure S35 – 1,2-Bis(6-iodo-2-methyl-1-benzothiophen-3-yl)hexafluorocyclopentene (**10**) HSQC NMR spectrum

Synthesis of 3,3'-(perfluorocyclopent-1-ene-1,2-diyl)bis(2-methyl-6-(acetylthio)benzo[b]thiophene) (13). A 100 ml two necked Schlenk tube, equipped with an argon gas inlet, a condenser and a magnetic stirrer was dried under vacuum, filled with argon and then charged with dried cyclopenthyl methyl ether(CPME) (20 mL). 1,2-Bis(6-iodo-2-methyl-1-benzothiophen-3-yl)hexafluorocyclopentene (10) (1.08 g, 1.45 mmol), CuI (10 mol %), 1,10- phenanthroline (20 mol %) and finally potassium thioacetate (2.17 mmol, 1.5 eq.) were added under argon and stirred at 100 °C for 18 h, monitoring by TLC (cyclohexane/AcOEt = 9:1, R_f = 0.153). The reaction mixture was cooled to room temperature and quenched with water (20 mL). The organic layer was separated and the aqueous layer was extracted with CPME (3 ×20 mL). The combined organic extracts were dried over $CaCl_2$, and the crude reaction mixture was purified, by silica gel column chromatography (gradient 100% cyclohexane-cyclohexane/AcOEt = 8.5:1.5), giving 0.68 g of 3,3'-(perfluorocyclopent-1-ene-1,2-diyl)bis(2-methyl-6-(acetylthio)benzo[b]thiophene) (6) (1.10 mmol, yield 76%) as a mixture of conformational isomers (parallel/antiparallel = 35:65).

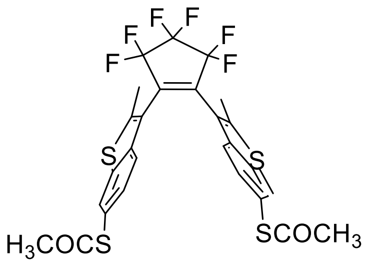

13p

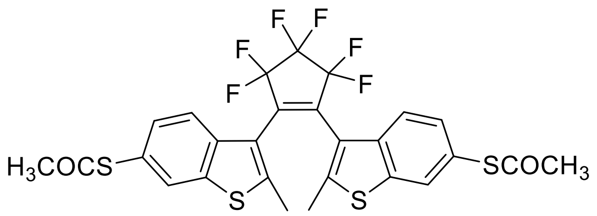

13ap

^{1}H NMR (400 MHz, $CDCl_3$) antiparallel conformer δ (ppm) 7.78 (s, 2H), 7.67 (d, J = 7.9 Hz, 2H), 7.38 (d, J = 8.2 Hz, 2H), 2.50 (s, 6H), 2.45 (s, 6H); parallel conformer δ (ppm) 7.70 (s, 2H), 7.54 (d, J = 8.5 Hz, 2H), 7.21 (d, J = 8.3 Hz, 2H), 2.39 (s, 6H), 2.23 (s, 6H). Parallel/antiparallel = 35:65.

^{13}C NMR (100 MHz, $CDCl_3$) antiparallel conformer δ (ppm) 193.97, 145.05, 138.95, 130.64, 128.35, 124.13, 122.61, 119.28, 30.23, 15.45; parallel conformer δ (ppm) 193.97, 144.56, 138.80, 130.47, 128.14, 122.36, 119.13, 30.23, 15.45.

^{1}H/^{13}C HSQC NMR ($CDCl_3$) antiparallel conformer δH/δC δ (ppm) 7.79/128.34, 7.67/122.60, 7.39/130.62, 2.50/15.53, 2.44/30.37 parallel conformer δH/δC δ (ppm) 7.70/128.14, 7.55/122.55, 7.21/130.6, 2.24/15.50, 2.38/30.34.

19F NMR (376 MHz, CDCl3) δ (ppm) -108.42 – -111.61 (m), -132.26 – -133.12 (m).

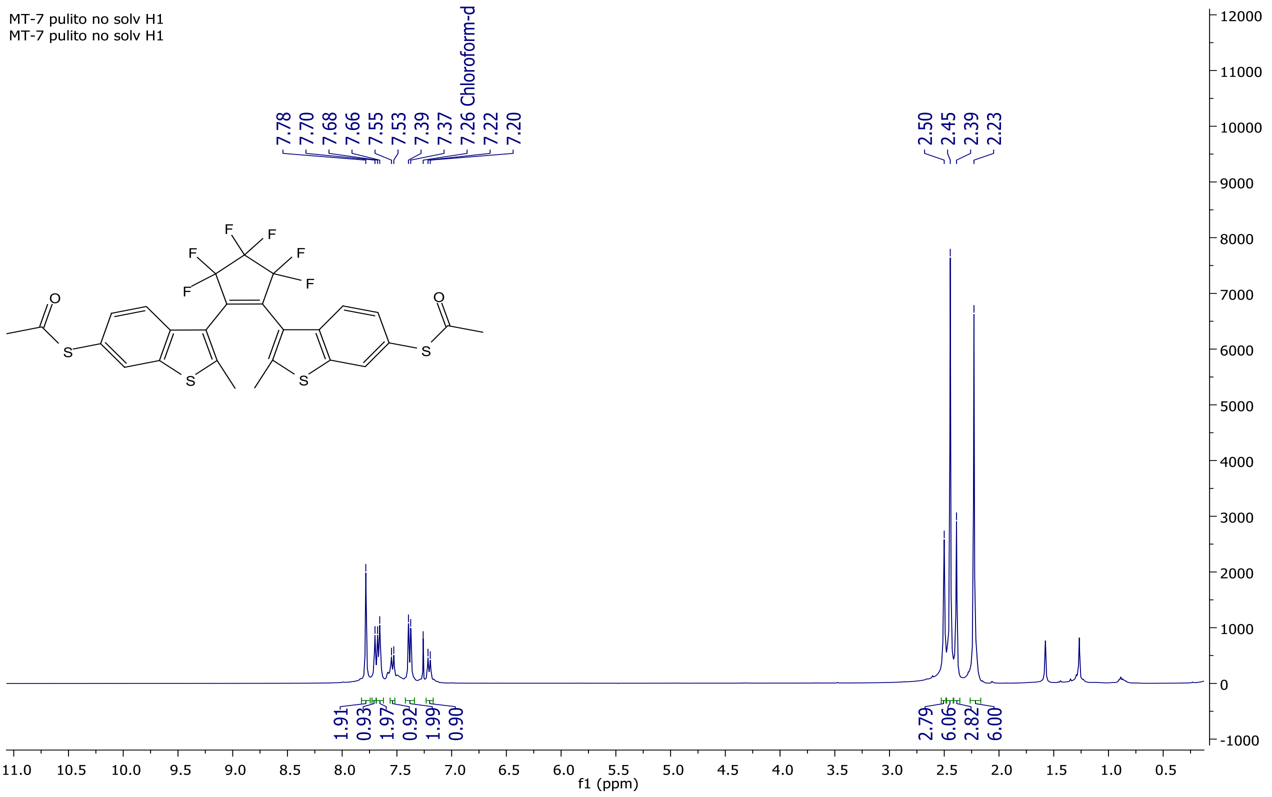

Figure S36 – 3,3'-(perfluorocyclopent-1-ene-1,2-diyl)bis(2-methyl-6-(acetylthio)benzo[b]thiophene) (**13**) ^{1}H NMR spectrum

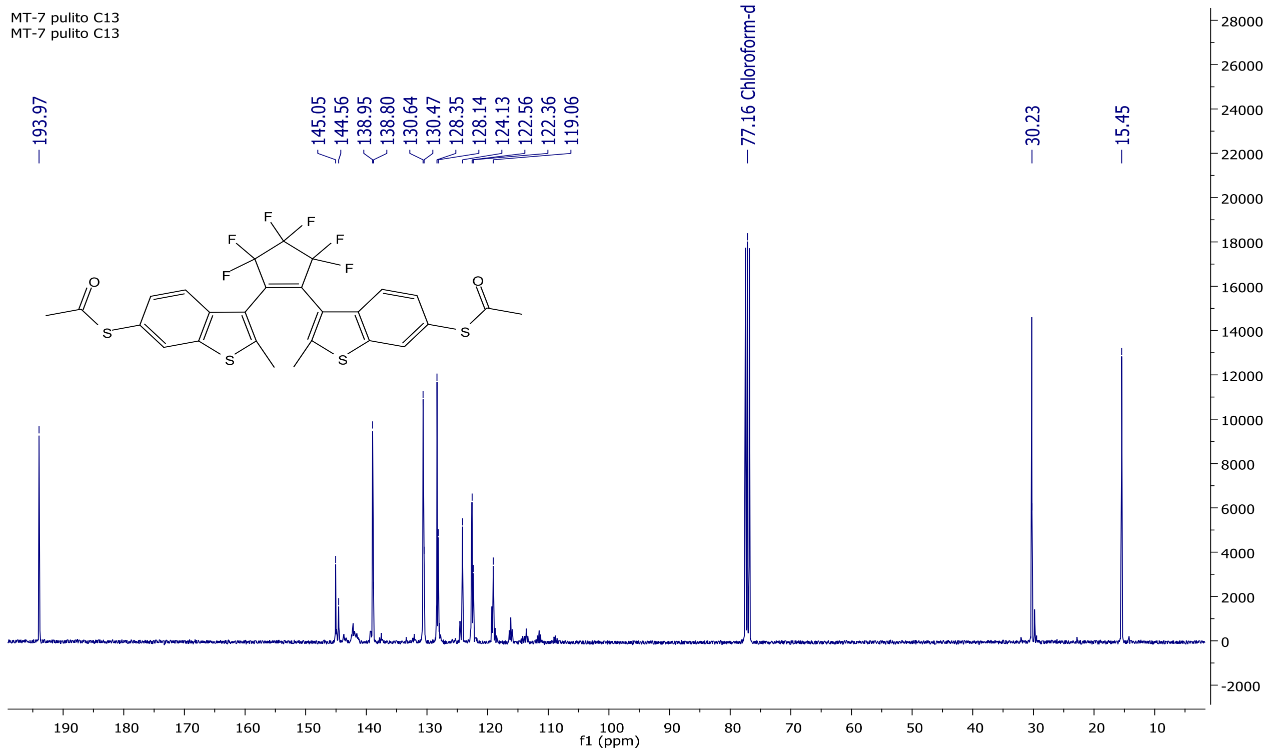

Figure S37 – 3,3'-(perfluorocyclopent-1-ene-1,2-diyl)bis(2-methyl-6-(acetylthio)benzo[b]thiophene) (**13**) ^{13}C NMR spectrum

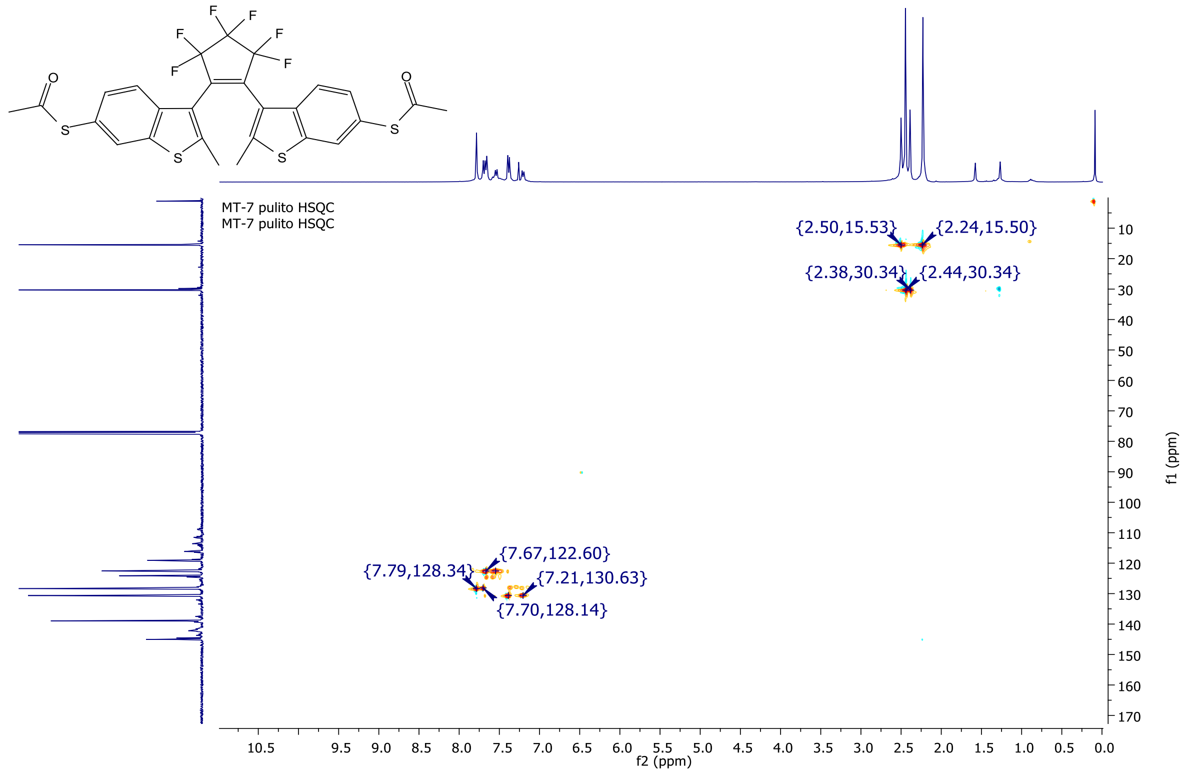

Figure S38 – 3,3'-(perfluorocyclopent-1-ene-1,2-diyl)bis(2-methyl-6-(acetylthio)benzo[b]thiophene) (**13**) HSQC NMR spectrum

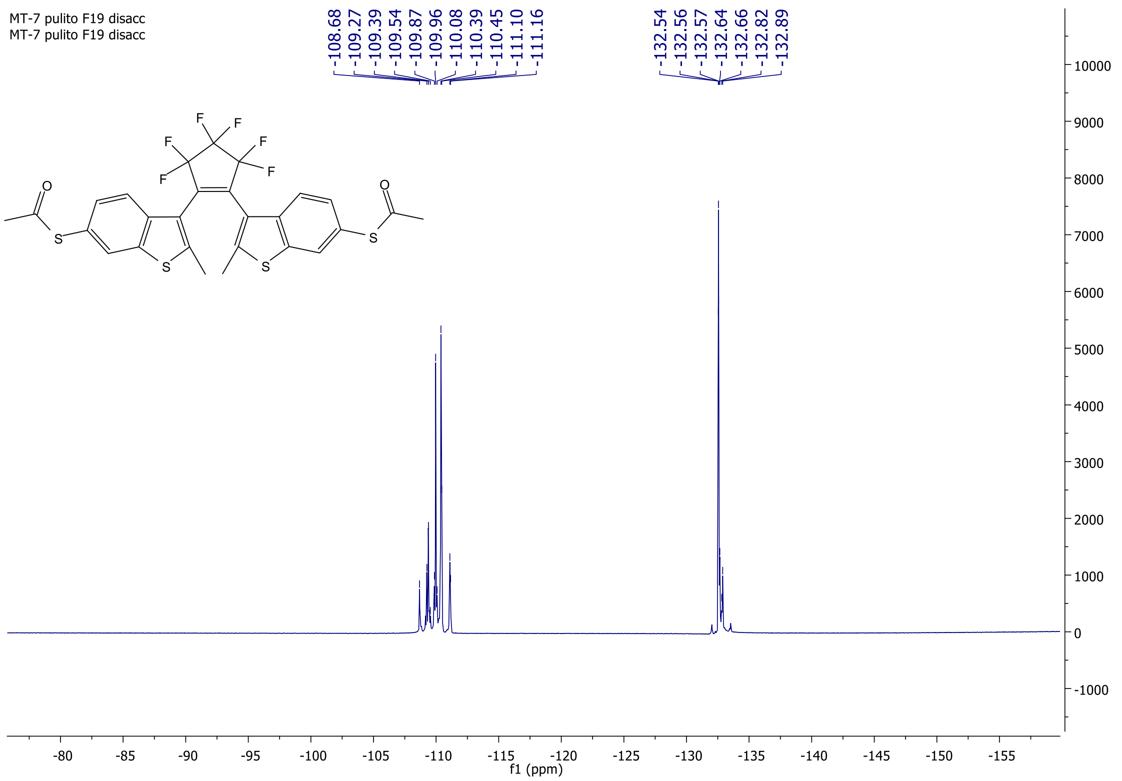

Figure S39 – 3,3'-(perfluorocyclopent-1-ene-1,2-diyl)bis(2-methyl-6-(acetylthio)benzo[b]thiophene) (**13**) ^{19}F NMR spectrum

---

[1] M. Barbero, I. Degani, S. Dughera, R. Fochi, *Synthesis* **2003**, *2003*, 1225–1230.

[2] F. Yang, G.-C. He, S.-H. Sun, T.-T. Song, X.-T. Min, D.-W. Ji, S.-Y. Guo, Q.-A. Chen, *J. Org. Chem.* **2022**, *87*, 14241–14249.

[3] T. C. Pijper, J. Robertus, W. R. Browne, B. L. Feringa, *Org. Biomol. Chem.* **2014**, *13*, 265–268.

[4] X.-Z. Yu, W.-L. Wei, Y.-L. Niu, X. Li, M. Wang, W.-C. Gao *Molecules* **2022**, *27*, 6232.

[5] a) Z. Zhang *et al. Dalton Trans.*, **2021**, 50, 4604-4612; b) M. Irie *et al. Chem. Eur. J.* **2001**, 7, 16, 3466-3473.
